# Lipid interactions couple turnercyclamycin antibacterial activity with carbohydrate-active enzyme packaging in membrane vesicles

**DOI:** 10.1039/d6cb00220j

**Published:** 2026-10-09

**Authors:** Bailey W. Miller, Albebson L. Lim, Mark T. Gasser, Zhenjian Lin, Daniel L. Distel, Eric W. Schmidt

**Affiliations:** a Department of Medicinal Chemistry, University of Utah Salt Lake City UT 84112 USA ews1@utah.edu; b Johns Hopkins Applied Physics Laboratory Laurel MD 20723 USA; c Ocean Genome Legacy Center, Northeastern University Nahant MA 01908 USA d.distel@northeastern.edu

## Abstract

Turnercyclamycins from shipworm-symbiotic bacteria are lipopeptides with promise for targeting bacterial infections. Their mechanisms of action and resistance suggest an essential role for lipid interactions. We purified analogs with lipid chain lengths ranging from C12 to C16 and tested them against *E. coli*, with and without Δ*mlaA*, which confers resistance to turnercyclamycin B but not to A. A C14 fatty acid provided the optimal side chain for activity against both strains. We disrupted a hydroxylating enzyme in *Teredinibacter turnerae* T7901, generated an unnatural analog, assigned specific activity to one of the tailoring enzymes, and genetically validated the *tur* biosynthetic pathway. By genome mining, we found structural homologs in *Gynuella sunshinyii* YC6258. Finally, we found that turnercyclamycins are selectively associated with *T. turnerae* membrane vesicles (MVs), linking these antibiotics to natural transport mechanisms. These results establish that lipid length determines turnercyclamycin antibacterial activity and suggest that the same interactions also govern ecological transport through MVs.

## Abstract 2

A tail of two functions: membrane vesicle association in symbiosis and antibacterial activity against pathogens.

## Graphical abstract

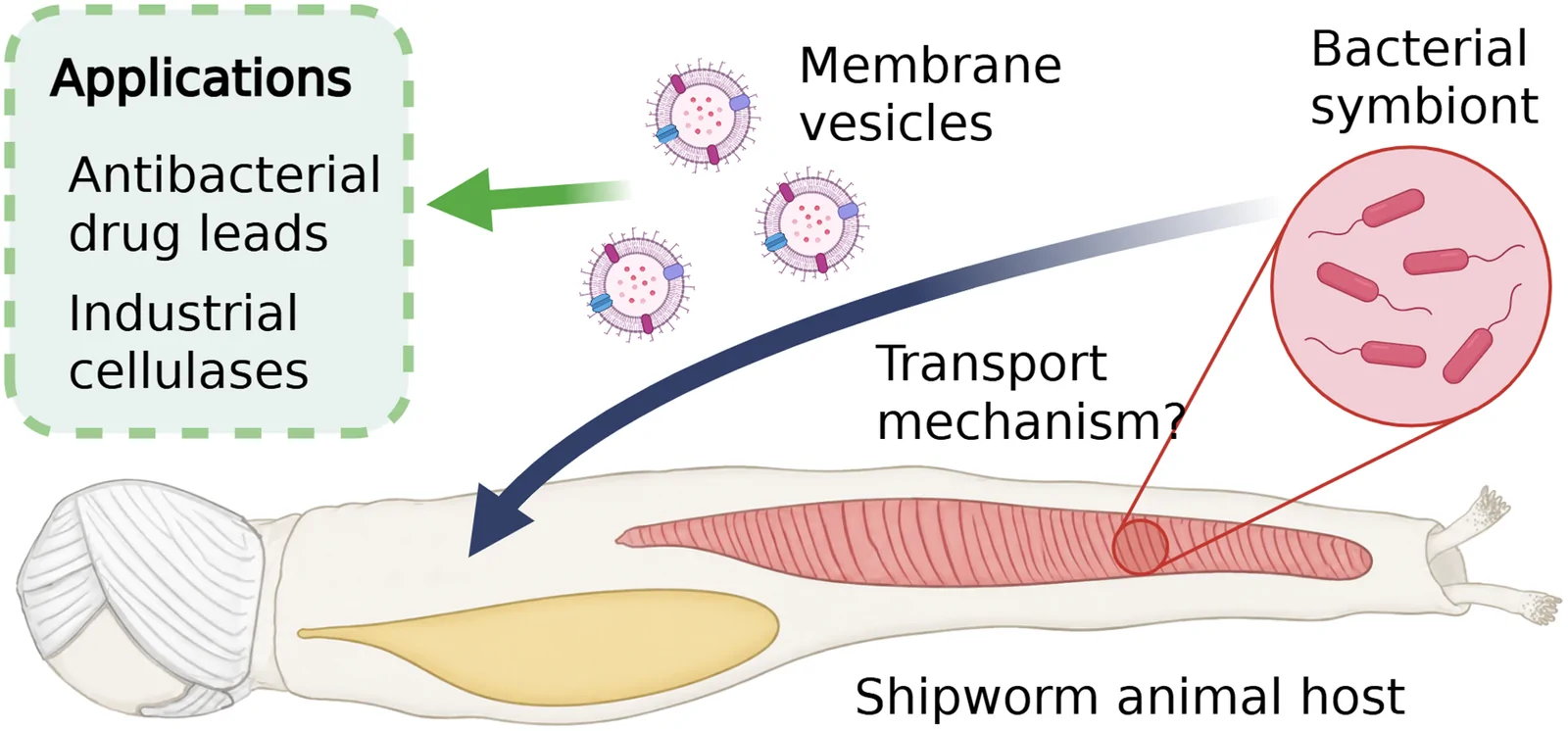

## Introduction

Shipworms are wood-eating clams that consume much of the wood in the world's oceans. Symbiotic bacteria, including *Teredinibacter turnerae*^1^ and related Gammaproteobacteria,^2^ assist these animals in degrading ingested wood particles using cellulases and other enzymes.^3^ In most cellulolytic symbioses, microbes live in the gut in direct contact with food particles, helping to degrade wood and also consuming the released sugars. Shipworms have found a different solution: the symbionts live in the gills, while the digestive cecum is relatively axenic.^4^ Remarkably, *T. turnerae* packages a wide range of wood-degrading (lignocellulolytic) enzymes in bacterial membrane vesicles (MVs), which are likely to be outer membrane vesicles (OMVs) (Fig. 1).^5,6^ We hypothesize that these MVs and their enzyme content play critical roles in the two-phase life cycle of these facultative symbionts, both in the environment and within the host's tissues. During the free-living phase, MVs enable *T. turnerae* to deliver a complex cocktail of hydrolytic enzymes to lignocellulosic substrates, thereby preventing dilution and ensuring the concomitant delivery of enzyme combinations that exhibit synergistic activity. Within the host, MVs may mediate the secretion and transport of symbiont-made enzymes from the symbiont-containing tissues in the gills to the digestive system *via* the ducts of Deshayes,^7^ which connect the gill bacteriocyte tissue to the gut. This separation of symbiont cells from the digestive system enables the host to access lignocellulose-derived sugars directly rather than the lower-energy metabolic products of gut microbes.

**Fig. 1:**
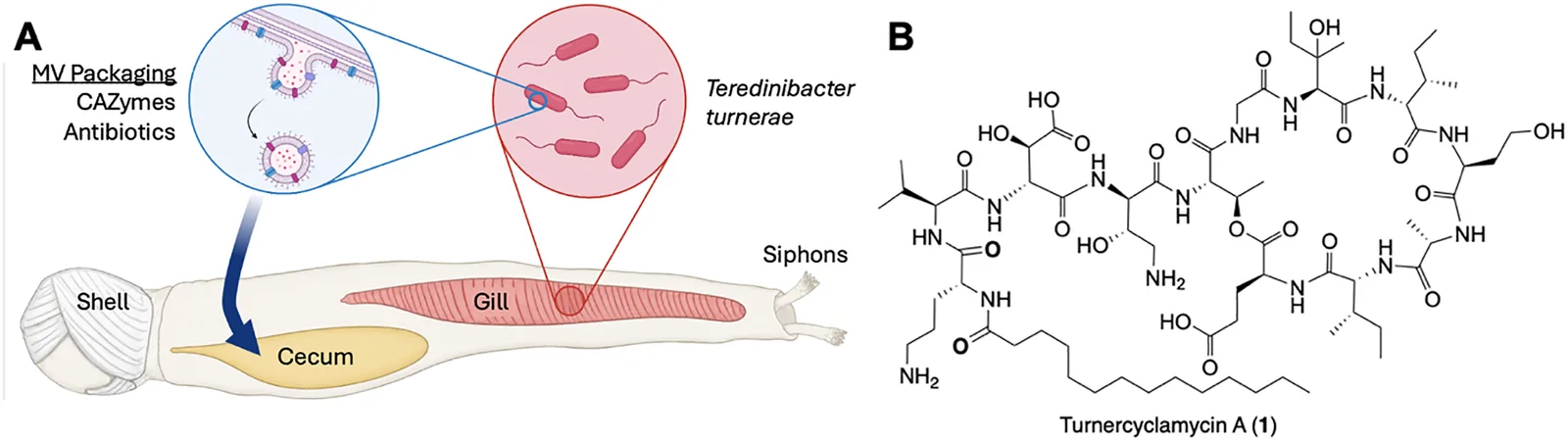
Overview of MV-mediated shipworm chemical ecology and turnercyclamycin structure. (A) *Teredinibacter turnerae* lives in dense colonies in specialized bacteriocytes in the gills where carbohydrate-active enzymes (CAZymes) and small molecules are packaged into MVs. These MVs may be transported to the cecum, where they aid in cellulose digestion and suppress competitive bacteria. (B) One of the effective antibiotics isolated from *T. turnerae* is the lipopeptide turnercyclamycin A (1).

In addition to producing many cellulases, *T. turnerae* and relatives are unusually rich in specialized metabolic pathways.^8^ Shipworm symbiont genomes have biosynthetic gene cluster (BGC) densities comparable to those of well-known antibiotic producers such as *Streptomyces*.^9^ One of these BGCs produces tartrolons, potent antibacterial and antiparasitic agents.^10^ After their isolation from *T. turnerae* cultures, tartrolons were also found in shipworm whole-tissue extracts, leading to the hypothesis that beyond producing cellulases for host nutrition, the symbionts use antibiotics to protect their intracellular niche and help keep the host's gut axenic.^11^ This idea, along with the prevalence of antibiotic pathways in the animal metagenomes,^8^ led us to propose that shipworm symbionts would provide unique, animal- and human-compatible antibiotics to address the microbial drug resistance crisis.

Based on this hypothesis, we previously isolated lipopeptides turnercyclamycins A (1) and B (2), which kill numerous multidrug-resistant Gram-negative bacterial pathogens and clear *Acinetobacter baumannii* infections in an animal model.^12–14^ Turnercyclamycins were isolated in part because they were proposed to be derived from the major BGC, *tur*, found in shipworm gill metagenomes from around the world. Before we elucidated the turnercyclamycin structures, we initially named this gene cluster family (GCF) GCF1, reflecting its importance as the most central and ubiquitous shipworm metagenome-encoded biosynthetic pathway, making its products priorities for isolation.^8^ While the *tur* cluster closely matches the predicted turnercyclamycin structures, experimental evidence firmly linking the compounds to the genes was still lacking.

Turnercyclamycins provide a bridge between MVs and antibiotic action, linking the fatty acyl chain to both ecology and potential therapeutic applications. We previously showed that exogenous MVs from *Escherichia coli* and specific exogenous lipids reduce or abolish the activity of these compounds.^14^ The turnercyclamycin B (2) fatty acyl chain differs from that of 1 by the presence of a *cis*-CH=CH bond, which greatly impacts bacterial resistance to the drugs.^13^ While turnercyclamycin A (1) antibacterial activity is unaffected by mutations to the MlaA lipid transporter, turnercyclamycin B (2) is inactive in the presence of some MlaA mutants. Further, turnercyclamycins disrupt lipid homeostasis in target bacteria. These and other data led us to hypothesize that turnercyclamycins cause this disruption by interfering with AcpP interactions at the metabolic intersection of phospholipid and lipopolysaccharide (LPS) biosynthesis.^14^ Because MlaA mutants selectively resist 2 but not 1, they provide a sensitive test strain for lipid-dependent structure–activity and structure-resistance relationships, in which the most active compounds are equipotent against wild-type and Δ*mlaA*.

We aimed to determine how the fatty acid side chain shapes turnercyclamycin activity. Through genome mining and improved culture and isolation conditions, we identified additional turnercyclamycin fatty acyl variants from *T. turnerae* and a closely related structural variant from the rhizosphere bacterium *Gynuella sunshinyii* (Fig. 2). Strikingly, nearly identical analogs with slightly shorter (C12 : 0, 3) and slightly longer (C16 : 0, 4) acyl chains were less active than the parent compound (C14 : 0, 1) against *A. baumannii* and *E. coli*; longer-chain 4 had the least activity, and it also lacked efficacy against Δ*mlaA* strains. In an attempt to obtain additional structural variants, we deleted an oxidase from the turnercyclamycin BGC (*tur*), eliminating the Asp β-hydroxy group from the peptide chain and adding experimental support for the BGC's predicted function. We showed that 1, 2, and 4, but not 3 or tartrolon, are present in *Teredinibacter* MV preparations, indicating a selective association of turnercyclamycins with MVs. Together, these observations suggest that variation in the lipid tail governs both antibacterial activity and transport, linking turnercyclamycin mechanism with its ecological context.

**Fig. 2:**
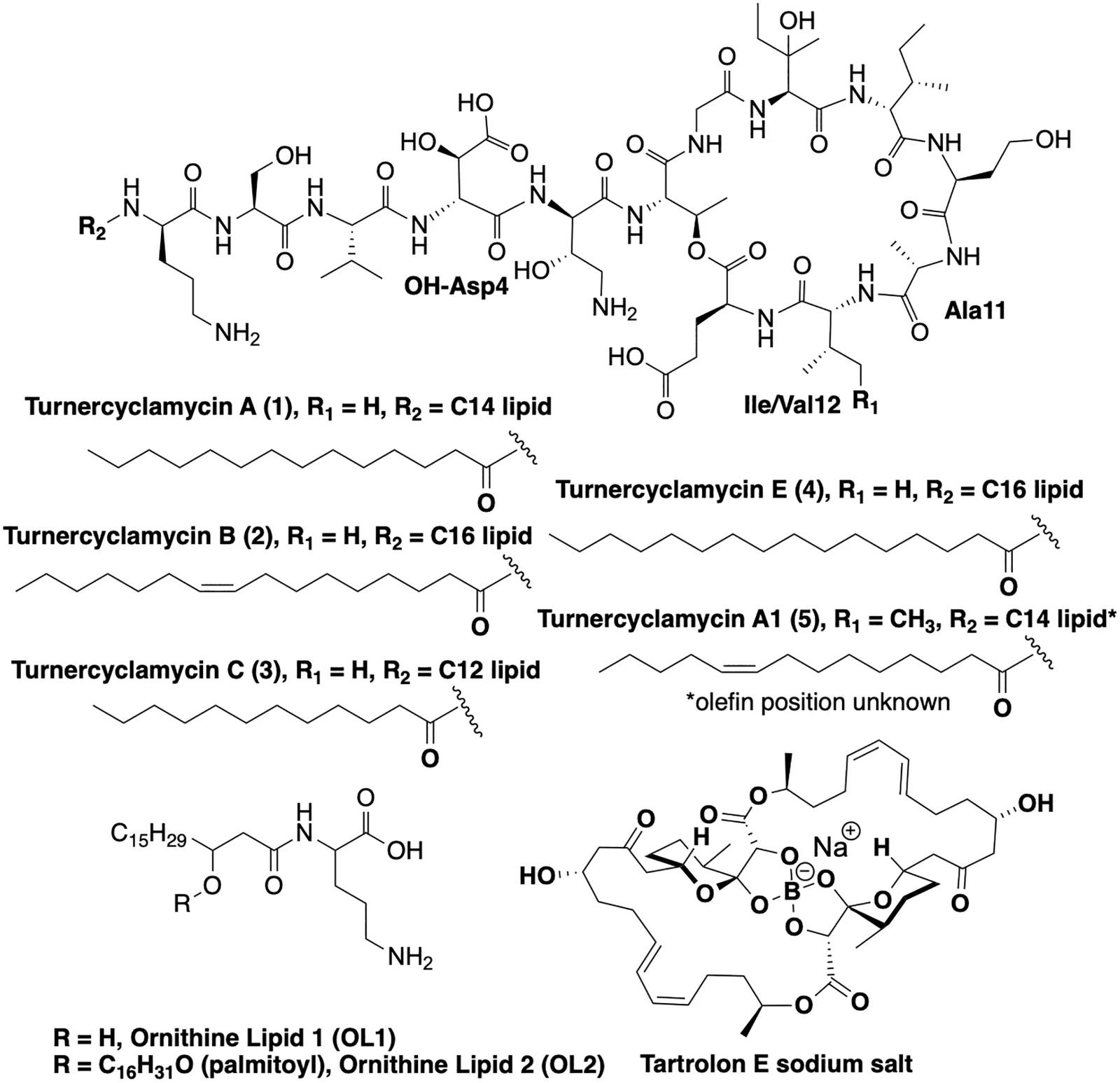
Compounds described in this study. Top compounds are antibiotics, turnercyclamycin variants from *T. turnerae* and *G. sunshinyii*. Bottom compounds include two ornithine lipids found in *T. turnerae* MVs and the antiparasitic and antibiotic tartrolon E from most *T. turnerae* strains.

## Results

### Turnercyclamycins and ornithine lipids selectively associate with *T. turnerae* MVs

Previously, Gasser and co-workers purified MVs from *T. turnerae* and demonstrated that they contained cellulases. Here, the same group used the same method to obtain MVs,^5^ which were then extracted and subjected to mass spectrometry targeting small-molecule metabolites. The *T. turnerae* MVs contained 1 and 2 (Fig. 1) as their major small-molecule components, with small amounts of 4 and no detectable amount of 3 (Fig. 3 and Fig. S1). In addition, we observed large quantities of ornithine lipids OL1 and OL2, with one and two acyl side chains, respectively. OL1 was recently characterized as *N*-3-hydroxyoleoyl ornithine and proposed to be racemic at the ornithine nitrogen.^15^ Intriguingly, the structures of these ornithine lipids closely resemble the tail end of turnercyclamycins, which also have ornithine in the d-configuration. Although we did not quantify relative metabolite concentrations across fractions, the MV metabolite profile did not simply mirror the cell pellet extract. Tartrolon E, the dominant metabolite in cell pellet extracts, was not detected in the MV fraction. These data support the selective association of turnercyclamycins with *T. turnerae* MVs rather than the nonspecific carryover of abundant cellular metabolites (Fig. 3A).

**Fig. 3:**
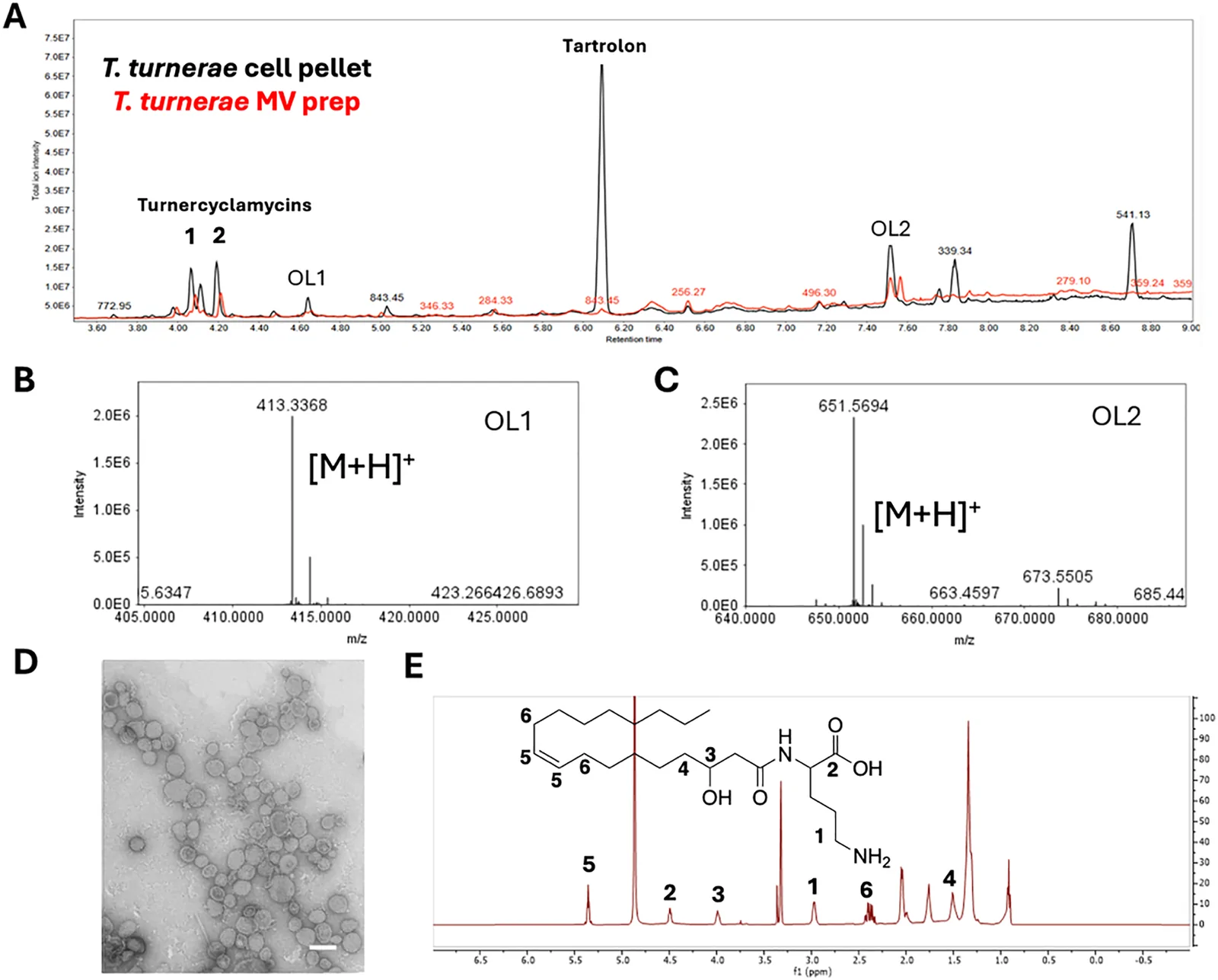
Turnercyclamycins A and B are specifically localized in *T. turnerae* membrane vesicles (MVs). (A) The total ion chromatograms (TICs) are shown for extracts from the *T. turnerae* cell pellet (black trace) and isolated MVs (red trace). Notably, tartrolon is highly enriched in cell extracts, but essentially absent from MVs, whereas the turnercyclamycins 1 and 2 and ornithine lipids OL1 and OL2 are found in both. The turnercyclamycin variants found in whole cells and MVs also differ (Fig. S1). High-resolution mass spectra for OL1 (panel B) and OL2 (panel C) are shown. (D) Microscopy confirms the purity of the MV preparation used (scale bar = 100 nm). (E) To confirm the identity of the ornithine lipids, OL1 was isolated, purified, and analyzed using the ^1^H NMR experiment shown, yielding a spectrum essentially identical to that of the previously reported *N*-3-hydroxyoleoyl ornithine.^15^

Ornithine lipids are associated with low-phosphate conditions, such as those used to produce turnercyclamycins in culture.^16^ In the specific case of turnercyclamycins, ornithine lipids may be significant because lipopeptide antibiotics are often described as cationic molecules that associate with negative membrane charge. Unlike those molecules, turnercyclamycins are net neutral. Ornithine lipids, which add positive charge to the membrane, thus may provide a favorable environment for turnercyclamycin binding. In addition, ornithine lipids are crucial components of some MVs, which confer properties such as protection against antibiotic action or interaction with the host immune system.^17^ Finally, the ornithine lipids discovered here are close homologs of the starter fatty acyl-d-ornithine substituents found in turnercyclamycins, suggesting a close biological and chemical relationship between these molecular species. Indeed, such lipids may be protective against diverse antibiotic compounds in ESKAPE pathogens including *A. baumannii*.^18^

The co-packaging of turnercyclamycins and cellulolytic enzymes in MVs reinforces the importance of GCF1/*tur* as a major pathway in shipworms and supports the hypothesis that symbiont-made antibiotics play a physiological role in host nutrition.

### Genetic validation of the *tur* BGC

Despite strong correlative evidence and firm logical support, the *tur* BGC has not been biochemically validated as the biosynthetic source of turnercyclamycins. To provide direct evidence, we attempted to knock out oxidases that are putatively involved in the synthesis of the three β-hydroxy amino acids. Despite extensive efforts, we did not obtain stable *turF* mutants through recombinant engineering; however, we successfully generated a scarless, in-frame Δ*turH* mutant (Fig. 4 and Fig. S2), which was validated by Sanger sequencing across the junction. Four knockout colonies were grown and extracted individually. For one of these, no turnercyclamycins were observed, whereas three produced a small amount of turnercyclamycin-like products that were lighter by 16 AMU, exactly consistent with loss of a single oxygen. This result provides strong evidence that the *tur* BGC is correctly identified as the turnercyclamycin biosynthetic pathway.

**Fig. 4:**
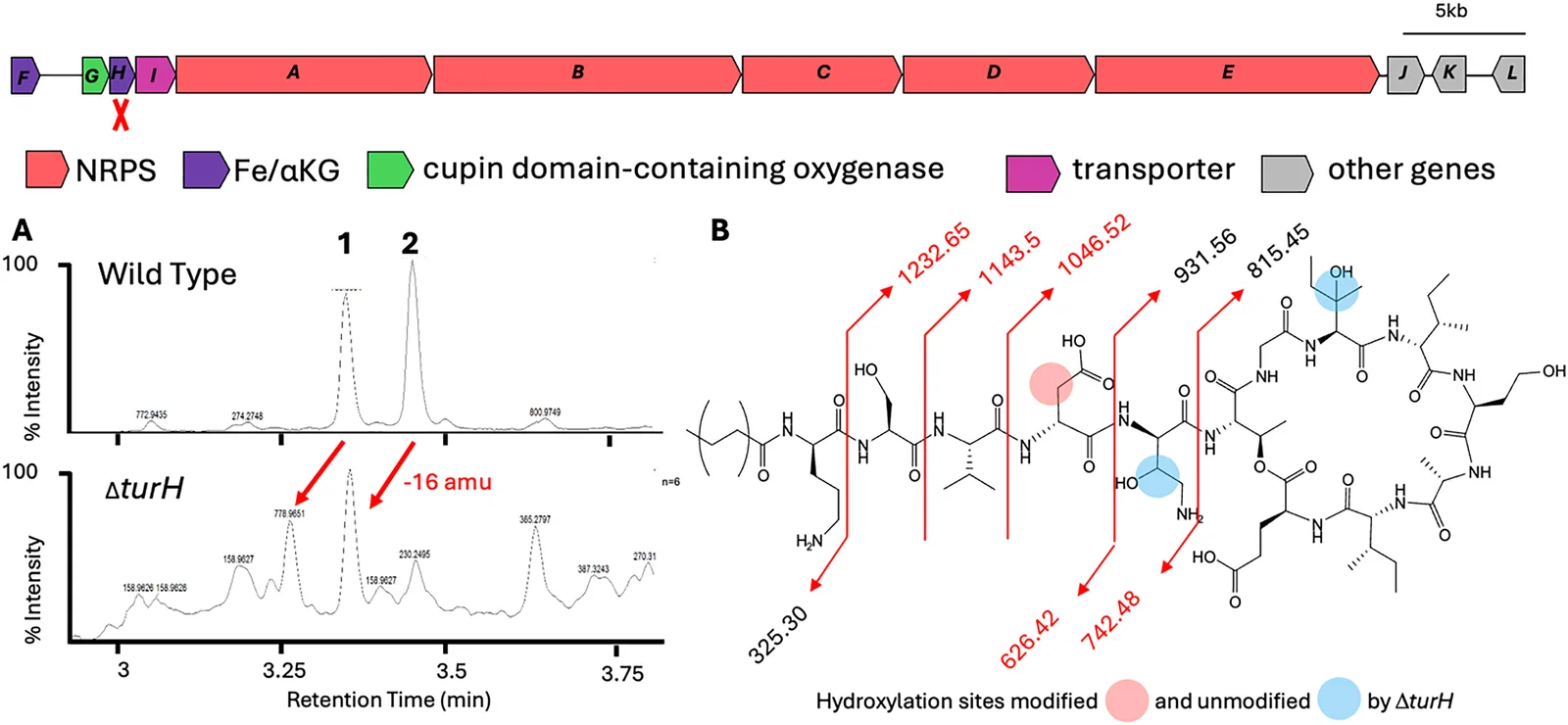
Deletion of *turH* abolished Asp4 hydroxylation in turnercyclamycins. The *Teredinibacter turnerae tur* biosynthetic gene cluster (BGC) diagram shows the site of a scarless, in-frame deletion with an “X”. (A) A comparison of the total ion chromatograms (TICs) of extracts from wild-type *versus* knockout strains reveals that turnercyclamycins A (1) and B (2) are replaced in the mutant by homologs that are lighter by 16 AMU, exactly consistent with loss of a single oxygen. Results from four independent replicate colonies are provided in Fig. S2. (B) A scheme of the MS^2^ data (full spectra of dehydroxy turnercyclamycins A and B are shown in Fig. S3 and S4) localized the missing oxygen to Asp4 (pink oval), but not to the other hydroxylation sites (blue ovals). As shown in the TIC, the *turH* knockout greatly decreased the production of turnercyclamycin analogs, which is why the non-turnercyclamycin peaks are comparatively much larger in the knockout.

Although fermentation of *T. turnerae*-Δ*turH* yielded only trace amounts of material, precluding purification, LC-MS/MS analysis and comparison of the resulting fragmentation patterns unambiguously localized the mass change to loss of oxygen from Asp4, while positions 5 and 8 remained intact. Together, these results established the role of TurH and provided the first direct experimental validation linking the *tur* BGC to turnercyclamycin biosynthesis.

### Discovery of new turnercyclamycin variants from *G. sunshinyii* YC6258

In previous work, we identified only one other bacterial species—the rhizosphere bacterium *Gynuella sunshinyii* YC6258 (RefSeq assembly accession GCF_000940805.1)—whose genome contained a putative *tur* BGC homolog (Fig. S2).^12^ Like the *T. turnerae tur* BGC, that of *G. sunshinyii* shared the putative oxidases *turH* and *turJ*, whereas only *T. turnerae* had putative oxidases *turF* and *turG*. Thus, it remained unclear which oxidases might be involved in turnercyclamycin tailoring. To address this issue, we compared turnercyclamycin production by *G. sunshinyii* YC6258 to that of *T. turnerae*. LC-MS/MS analysis indicated that a series of related molecules was present in the cell pellet extract, including one major compound and several minor analogs. Isolation of the major product yielded the previously unreported metabolite, turnercyclamycin A1 (5). MS/MS analysis revealed that the *G. sunshinyii* molecules differed from those found in *T. turnerae* in their peptide backbones, with the major product 5 showing a −14 AMU mass difference (Fig. 5). MS^2^ fragmentation localized this difference to the 8-amino-acid macrocycle but was insufficient to determine which amino acid position was altered; fortunately, a full NMR dataset could be obtained (Fig. S5–S11 and Table S2). The HSQC NMR data for 1 and 5 were superimposable except for a significant change at position 12 and minor changes at amino acids 11 and 13; the resulting difference was confirmed by COSY NMR to be due to a substitution of *allo*-Ile in 1 for Val in 5. The almost identical proton and carbon chemical shift data for the compounds at all other positions strongly suggested that the peptide chains are otherwise identical in both sequence and configuration. In addition, MS^2^ and NMR data revealed a minor difference in the lipid side chains: both 1 and 5 have C_14_ fatty acids, but 5 also contains an olefin at an undefined position.

**Fig. 5:**
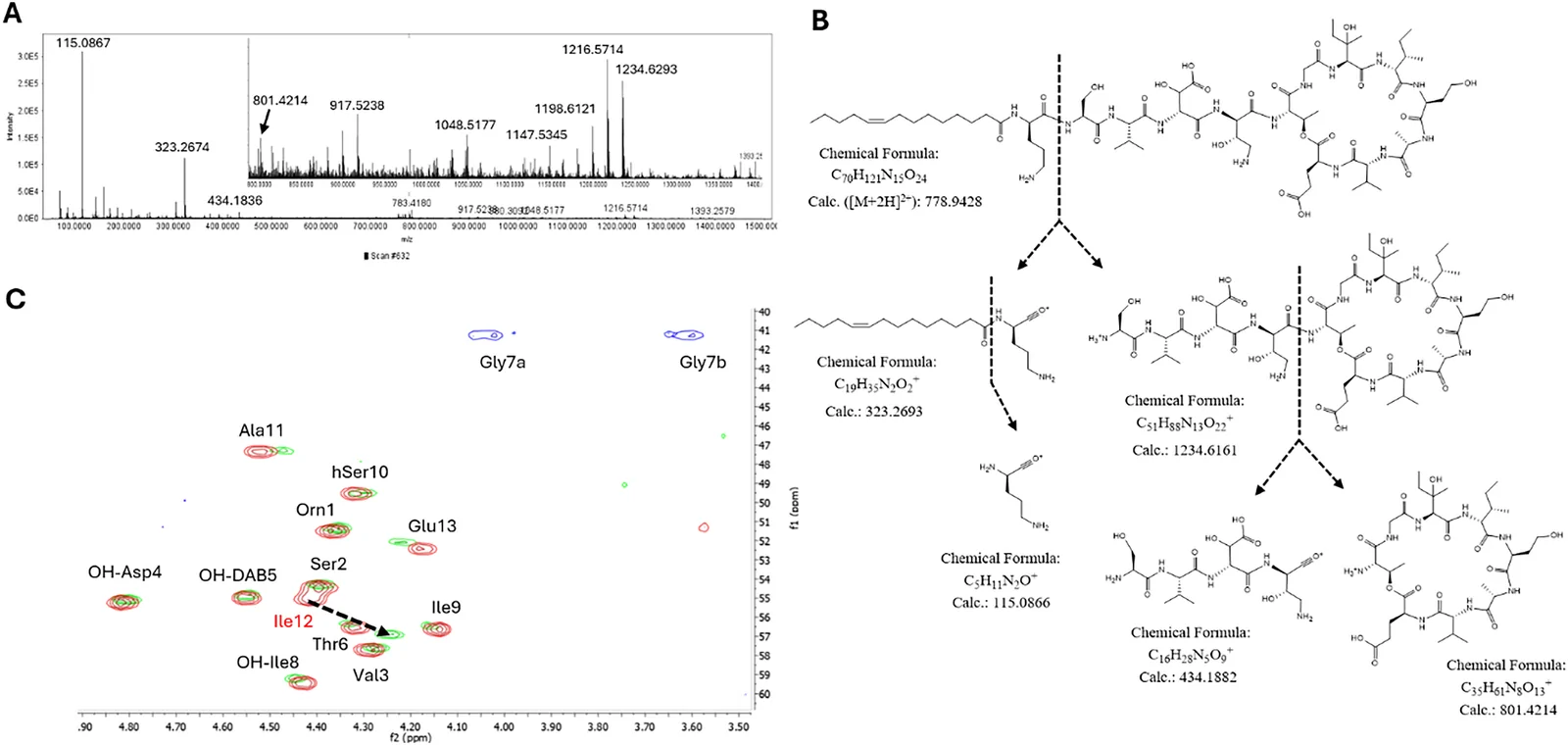
*Gynuella sunshinyii* produces turnercyclamycin A1 (5), an Ile12-Val variant with a slightly modified lipid chain. (A) MS^2^ spectra of the purified compound and comparison with those for previously characterized turnercyclamycins reveal (B) a fragmentation pattern in which an acyl-ornithine fragment is ejected, which further fragments to release ornithine (*m*/*z* 115.1). The fragment at *m*/*z* 801.4 indicates that the site of derivatization compared to *Teredinibacter*-derived turnercyclamycins is in the cyclic portion of the peptide. (C) Overlay of the alpha proton region of the HSQC NMR spectra for turnercyclamycins A (1) and A1 (5) reveals a shift in the alpha proton for amino acid 12, which is an Ile in *T. turnerae* peptides.

Although yields precluded NMR characterization of minor analogs, LC-MS/MS analysis indicates additional peptide backbone modifications (Fig. S12). One minor constituent shows an additional −14 AMU mass difference relative to 5 (−28 AMU from 1) in the peptide macrocycle. Another analog shows fragments consistent with a −30 AMU mass difference relative to 5, indicating loss of a methyl group and an oxygen atom. These arise from an additional Ile-to-Val substitution at either position 8 or 9, followed by loss of the beta-hydroxyl group at position 8.

Given that TurH selectively hydroxylates Asp, it was initially surprising that the oxidase differences encoded by the *T. turnerae* and *G. sunshinyii tur* BGCs did not lead to structural differences in the resulting products. Homologs of the oxidases were found outside of the *G. sunshinyii tur* BGC (Fig. S13), suggesting either that these unclustered homologs might be involved in the biosynthesis of 5 or that oxidases such as TurF might be involved in other pathways, such as central metabolism.

### Isolation of additional turnercyclamycin variants from *T. turnerae*

Given the purification challenges described above, we made methodological improvements to purify minor analogs previously detected by MS in *T. turnerae* extracts. While developing methods to improve the yield of tartrolon E from *T. turnerae* T7901^19^ we also substantially improved the process for obtaining purified turnercyclamycins. With a streamlined method in hand, we isolated the minor derivatives turnercyclamycins C (3) and E (4) in sufficient quantities for structure elucidation and biological assays. Based on NMR and MS^2^ experiments (Fig. S14–S20), we determined that the newly isolated compounds were identical to 1 and 2, except that they contained different fatty acids—lauric and palmitic acids, respectively. The small amounts of these compounds obtained precluded accurate measurement of optical rotation, but for 1 and 2 we measured [*α*]_D_^20^ = −5.2° and −6.2° (*c =* 5 mg mL^−1^, CH_3_OH), respectively. We could not isolate turnercyclamycin D, previously suggested by MS data, in sufficient yield and purity for NMR or assays.^12^ In addition, we purified turnercyclamycin A butyl ester 1b and determined its structure using the same methods (Fig. S22–S26). We further localized the ester to OHAsp4 based on a NOESY correlation and changes in the residue's chemical shift values. We hypothesize that this compound is an isolation artifact, as butanol was used to prevent excessive foaming in turnercyclamycin-rich extracts that also contain TFA. The ester 1b did not kill bacteria in assays.

### Acyl chain governs antibacterial activity and resistance in turnercyclamycins

In previously reported broth dilution assays,^12–14^ we showed reproducible MIC_90_s of 8 µg mL^−1^ for 1 and 2 against *A. baumannii* and 1 µg mL^−1^ against *E. coli* (or 2 µg mL^−1^ against some strains). While 1 retains potency against Δ*mlaA* disruptants, the activity of 2 is completely abolished.

We tested compounds 1b and 3-5 against *A. baumannii* and *E. coli* strains C600 and BW25113 (Fig. 6 and Fig. S27, S28). Compounds 3 and 4 were less active than 1 and 2 against wild-type strains. Turnercyclamycin C (3), with a shorter lipid chain, had MIC_90_s that were 2-fold higher against *A. baumannii* and 4-fold higher against *E. coli*. We observed much lower activity of the longer-chain turnercyclamycin E (4) than that of 1 or 2, with MIC_90_s > 32 µg mL^−1^ against *A. baumannii* and 8 µg mL^−1^ against *E. coli* strains. Compound 5 from *G. sunshinyii*, the most similar compound to 1 in lipid length, yielded an MIC_90_ against *A. baumannii* of 8 µg mL^−1^, identical to that of 1. Because of the difficulty of obtaining sufficient material from *G. sunshinyii* and the similarity in activity, we performed no further tests beyond an initial assessment.

**Fig. 6:**
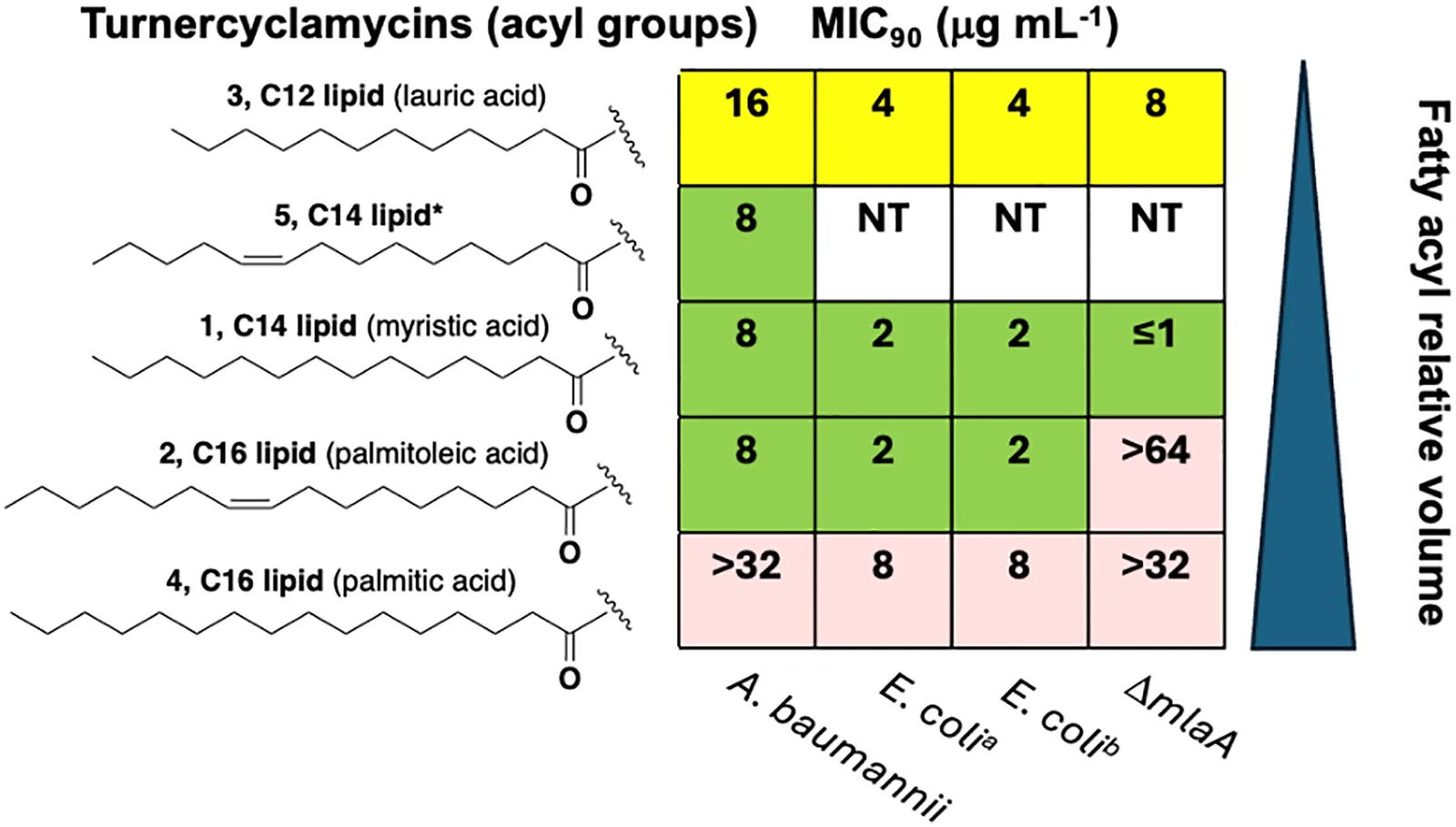
Activity of turnercyclamycin variants. Turnercyclamycins with different lipid tails were isolated and tested in broth dilution assays against *Acinetobacter baumannii*, *Escherichia coli* C600^a^, and *E. coli* BW25113^b^ from the Keio collection. In addition, to test the *mlaA* mutant that confers resistance to 2 but not to 1, *E. coli* BW25113 Δ*mlaA* from the Keio collection was used. Results (reported as the minimal inhibitory concentration to stop 90% of growth, or MIC_90_) for 1 and 2 represent previously reported, highly reproducible data, while those for 3, 4, and 5 are reported here for the first time. Green indicates the highest activity, while red indicates a significant loss of activity. Yellow is intermediate. The * for compound 5 indicates that the position of the double bond in the C14 lipid has not been determined. > indicates the maximum dose tested.

We further evaluated compounds 3 and 4 against the *E. coli* BW25113 Δ*mlaA*. Compound 3 was still active against the *mlaA* mutant, but with a reduced potency of 8 µg mL^−1^, while that of 4 was essentially abolished (MIC_90_s >32 µg mL^−1^).

An interesting observation from this test series was that although bacterial growth inhibition of over 90% (MIC_90_) could not be achieved, we often observed a ∼50% decrease in bacterial growth at the assay endpoint (an apparent MIC_50_). For example, compound 4 and butyl ester 1b exhibited relatively potent MIC_50_s despite failing to inhibit growth beyond that point. We had previously noted this effect with some turnercyclamycin analogs under certain conditions, such as when tested with *Staphylococcus aureus*. We further investigated this partial resistance using light microscopy (Fig. S29). When *E. coli* BW25113 Δ*mlaA* was treated with 2 or 4, filamentous forms of *E. coli* were observed, suggesting a deficit in septation despite the lack of cell killing. This is likely related to drug-induced modification of lipid homeostasis.

In summary, none of the analogs is more effective than the originally discovered turnercyclamycins A and B (1 and 2) at killing *A. baumannii* or *E. coli*, and most are less effective. Even minor changes in lipid length and olefin content led to drastic changes in activity. Myristic acid (C14) appears to be the most effective, yielding the potent compound 1, which is not affected by mutations in *mlaA*, whereas truncating or elongating the chain results in a rapid decrease in activity. Additionally, at least one substitution in the amino acid sequence is tolerated without loss of activity, suggesting that synthetic analogs of those amino acids should be explored. Finally, the observation that activity is blocked by a butyl ester suggests that the OH-Asp residue likely engages in specific interactions that mediate drug pharmacology, such as binding to the protein target or setting the charge state required for membrane interaction.

## Discussion

In this investigation, we experimentally link the *tur* BGC to turnercyclamycin production for the first time. This BGC is a core component of the genomes of all *T. turnerae* strains identified to date, indicating that it is essential to the survival of this facultatively symbiotic bacterium.^8^*T. turnerae*, in turn, is nearly ubiquitous among shipworm metagenomes, demonstrating its essential role in host survival. Taken together, these observations support the hypothesis that turnercyclamycins play an important functional role in a widespread form of symbiosis found in nearly all shipworm species throughout the world's oceans. As shipworms are the major wood-degrading animals in marine environments, they cause billions of dollars in damage to ships and marine structures, but also play a major role in linking terrestrial and marine carbon cycles.^20^ Thus, our work suggests that turnercyclamycins have broad ecological impacts on marine ecosystems.

To exert their effects both in nature and as therapeutic agents, antibiotics require potency against their target and a delivery method to reach it. Turnercyclamycins have both: the major compounds kill challenging bacterial pathogens, and they are packaged and potentially delivered in MVs. Indeed, bacterial MVs are increasingly studied as natural transporters of antibiotics, as natural drug delivery systems, and as mechanisms of resistance in microbial interactions.^21^ In various contexts, bacterial MVs are described as either promoting or inhibiting antibacterial activity in both artificial and natural settings.^22^ We show that, along with cellulases, turnercyclamycins are specifically associated with MVs produced by *T. turnerae*, suggesting a potential mode of targeted delivery for these molecules in both the free-living and symbiotic phases of *T. turnerae's* life cycle.

While the mechanism underlying MV association remains unknown, we hypothesize that by co-packaging these two types of molecules, *T. turnerae* simultaneously supplies its host with lignocellulolytic enzymes to aid in wood digestion and a potent antibiotic to help maintain the cecum free of bacteria that could compete with the host for wood-derived sugars. Similarly, during its free-living stage, co-packaging within MVs could allow *T. turnerae* to deliver a functional selection of lignocellulolytic enzymes in effective stoichiometric ratios to wooden substrates with minimal dilution, while at the same time decreasing competition from other microorganisms for the soluble products of their digestion. This integrated ecological strategy, first demonstrated here in *T. turnerae*, suggests that MV fractions may be a high-priority source for discovering and identifying potent antibiotics—an exploratory approach that may be applied to many environmental bacteria.

Once the antibiotic reaches its target, it must be potent enough to halt growth or kill the target bacteria. The major turnercyclamycins potently kill several Gram-negative bacterial species, and here we show that this killing is highly dependent on lipid side-chain length. While therapeutic lipopeptides usually exhibit an optimal lipid length,^23^ they are often shorter-chain than the active turnercyclamycins. Much of the literature suggests that lipopeptide action is based on binding to and weakening or breaking the bacterial cell membrane, and that optimum lengths are tailored for this activity. Based on a series of studies, we previously proposed that turnercyclamycins do not act *via* this mechanism, but instead directly target specific proteins, such as transporters or potentially AcpP-binding proteins that modify C14 lipids.^14^ Our work supports this hypothesis, as activity quickly drops off for non-ideal lipids. This is not just about pure chain length, but also about volume. For example, compound 2 contains 16 carbons, but its olefin makes it effectively more compact than the saturated, 16-carbon 4.

In addition to confirming the *T. turnerae tur* cluster's function, the biosynthetic work here links the hydroxylating enzyme TurH to the specific hydroxylation of the Asp4 residue. Based on the enzyme's predicted sequence, we anticipate that it will likely hydroxylate aspartic acid on an intermediate covalently tethered to the NRPS, similar to what the Butler group has described in siderophore biosynthesis.^24,25^ This could explain the greatly decreased titer in the *turH* knockout mutants, although other possibilities remain. What was surprising to us was that the *G. sunshinyii* product turnercyclamycin A1 (5) showed the identical oxidation pattern, despite the fact that the *G. sunshinyii tur* cluster contained a different set of oxidases than that in *T. turnerae*. The consistent presence of *turH* suggests that the enzyme is responsible for Asp4 hydroxylation in both cases, but further understanding of turnercyclamycin biosynthesis awaits additional studies.

This study has several limitations. Primarily, fermentation products were isolated in small amounts in some cases, limiting downstream experiments, especially given the notorious difficulty of separating lipopeptides with minor differences in lipid chain length. In addition, some of the inactive antibiotics tested here may be more active against species or strains we did not test. Although our work demonstrated the activity of 1 and 2 in many other bacterial contexts, we have yet to determine the full spectrum of their action and that of related molecules. Finally, it will be necessary to quantitatively assess the tissue distribution and effective concentration of these molecules in their shipworm hosts, particularly in the wood-digestion organ.

In summary, this work links two distinct and critical fields: the development of new compounds to combat the antibiotic resistance crisis and the ecophysiology of antibiotic use by animals and their symbiotic bacterial partners in natural settings. Here, we show that one of the most prevalent antibiotics in the symbiotic bacteria of living shipworms worldwide is sequestered within MVs. These MVs are also loaded with bacterial wood-degrading enzymes, enabling the animals to consume wood in the sea. This natural delivery method has implications for human therapy, as evidenced by analysis of antibiotic components that dictate both MV localization and effective treatment of lethal, drug-resistant human pathogens. Understanding the basic biology of animals and their symbionts is thus critically important for priority economic and health applications.

## Materials and methods

### General experimental procedures

NMR data were collected on a Varian 500 MHz spectrometer equipped with a 5 mm Varian HCN Oneprobe (^1^H 500 MHz, ^13^C 125 MHz). Data were analyzed in MestReNova (Mestrelab Research). Residual solvent signals were used for referencing. High-resolution mass spectrometry data were collected using a Waters Xevo G2-XS qTof. The ion source was a Waters Zspray ESI, and chromatography was performed using a Waters Acquity H class UPLC system equipped with a Waters Acquity HSS T3 column (2.1 × 100 mm, 1.8 µm). HPLC analysis and semi-preparative scale purification were completed on a Dionex Thermo UltiMate 3000 system equipped with an autosampler, fraction collector, and diode array detector. Analytical chromatography was performed using a Phenomenex Luna C18 column (4.6 × 150 mm, 5 µm) and semi-preparative HPLC was performed using a Phenomenex Luna C18 column (10 × 250 mm, 10 µm). Optical rotations were measured on a PerkinElmer PE-343 polarimeter using a final concentration of 5 mg mL^−1^ in MeOH.

### Preparation/purification of MVs

*T. turnerae* strain T7901 (ATCC 39867; GenBank CP001614) was selected for MV preparation. MVs were prepared as in Gasser *et al.*^5^ Briefly, bacterial cells were separated from the culture supernatant by 2 sequential centrifugation steps at 5000 × *g* for 20 min at 4 °C. The final supernatant was filtered through a 0.22 µm polyethersulfone filter. MVs were then pelleted from the filtrate by ultracentrifugation at 120 000 × *g* (T-647.5 rotor, Sorvall) for 90 min at 4 °C, followed by resuspension in 0.1 M phosphate-buffered saline (PBS) and fractionation by bottom-up density gradient ultracentrifugation. MVs were resuspended in 60% iodixanol density gradient medium (Optiprep) to a final density of 45% (w/v) and overlayered with 40%, 35%, 30%, 20%, and 10% iodixanol layers, followed by a final layer of 0.25 mL of 0.1 M PBS. After centrifugation (16 h, 150 000 × *g*, 4 °C, SW55 Ti rotor, Beckman-Coulter), MVs were collected at the interface of the 30% and 20% layers. Iodixanol was removed by passive diffusion dialysis (1000 kDa MWCO) in 50 mM ammonium bicarbonate, pH 8.3, at 4 °C, and the MV-containing fraction was vacuum concentrated to 100 µL (SPD121P SpeedVac, Thermo Scientific).

### Metabolomic analysis of *T. turnerae* cell pellets and MV preps

*T. turnerae* cell pellets were collected and extracted as described above. Extracts were diluted to 10 ppm in LC-MS-grade methanol and analyzed by LC-ESI-HRqTOFMS. *T. turnerae* T7901 MV prep (40 µg) was transferred to a microcentrifuge tube and lyophilized to dryness. MeOH (100 µL) was added to the dried preparation, which was vortexed and extracted for 30 min. 50 µL was decanted with a micropipette, then centrifuged at 13 000 × *g* for 5 min to pellet particulates. 1 µL was injected for LC-MS analysis.

### Plasmid construction

A recombinant plasmid for allelic exchange was constructed using Gibson assembly. The suicide vector pCM433^26^ served as the plasmid backbone. An 824 bp region upstream and a 720 bp region downstream of the target gene were PCR amplified from genomic DNA. Primers were designed with overlapping sequences complementary to adjacent fragments and to the linearized pCM433 backbone to facilitate Gibson assembly. The assembled plasmid was transformed into *Escherichia coli* DH10B, and correct assembly was verified by Sanger sequencing. Verified plasmids were purified and subsequently transformed into *E. coli* S17 for conjugative transfer.

### Gene disruption in *T. turnerae*

The recombinant pCM433-derived plasmid was introduced into *Teredinibacter turnerae* T7901 *via* conjugation with *E. coli* S17 as the donor strain. First-round homologous recombination events were selected on kanamycin-containing medium. Kanamycin-resistant colonies were passaged twice in nonselective medium to allow resolution of the integrated plasmid through a second homologous recombination event. Cultures were then plated on sucrose-containing medium to counterselect cells that retained plasmid sequences. Colonies that regained kanamycin sensitivity were selected as candidates that had excised the plasmid backbone and undergone allelic exchange. Positive colonies were confirmed by sequencing.

### Analysis of *tur* clusters

The genome assembly of *G. sunshinyii* was downloaded from NCBI, and BGC prediction was performed using the online antiSMASH 8.0 webtool. The *tur* BGC from *G. sunshinyii* was extracted and compared with the *tur* BGC from *T. turnerae* using clinker.^27^ The TurH and TurF amino acid sequences were used as queries in a BLASTp search against the genomes, and the protein sequence hits were aligned using T-Coffee.^28^ The phylogenetic tree was built using IQ-TREE.^29^ Similarly, TurG was used as a biosynthetic hook to mine for the cupin domain-containing oxygenase in the two genomes.

### Bacterial culture

*Teredinibacter turnerae* T7901^30^ was revived from glycerol stocks on SBM agar supplemented with cellulose (0.2%) and incubated at 30 °C. Single colonies were used to inoculate 50 mL of SBM broth in 125 mL Erlenmeyer flasks and cultured for 24 h at 30 °C with shaking at 100 rpm to generate seed cultures. Production cultures were prepared in Fernbach flasks containing 1 L of Instant Ocean-based SBM. Flasks were inoculated with 1 mL of seed culture and incubated at 30 °C with shaking at 100 rpm for 4 days.

*G. sunshinyii* YC6258^31^ was obtained from NBRC (Strain ID 109345). It was revived from glycerol stocks on marine agar and incubated at 30 °C. Single colonies were used to inoculate 7 mL of marine broth cultures in glass culture tubes and incubated overnight at 30 °C with shaking at 170 rpm. Production cultures were prepared in Fernbach flasks (2.8 L) containing 1 L of marine broth. Flasks were inoculated with 1 mL of seed culture and incubated at 30 °C with shaking at 170 rpm for 4 days.

### Harvest and extraction of bacterial cultures

Cultures were harvested by centrifugation at 7068 × *g* for 20 min. Cell pellets were collected, frozen at −80 °C, and lyophilized to dryness. Dried biomass was pulverized and extracted three times with MeOH containing 0.1% TFA. The first extraction included sonication followed by an overnight extraction at room temperature, whereas subsequent extractions were performed for at least 2 h at room temperature. Combined extracts were filtered and concentrated *in vacuo*.

The crude extract was partitioned between H_2_O and EtOAc. Material accumulating at the solvent interface was collected and re-extracted twice from the aqueous phase. Combined interface fractions were concentrated under reduced pressure and dissolved in MeOH containing up to 1% TFA, then filtered through PVDF syringe filters prior to purification.

### Turnercyclamycin purification

Filtered interface fractions were purified by semi-preparative HPLC using a Phenomenex Luna C18 column (10 × 250 mm, 10 µm). Mobile phase A consisted of Milli-Q H_2_O containing 0.05% TFA, and mobile phase B consisted of acetonitrile. The chromatographic method employed an initial isocratic hold at 30% B for 2 min, followed by a linear gradient to 55% B over 12 min, a 1 min hold at 55% B, and re-equilibration to 30% B over 5 min. Compounds 1, 2, 3, and 4 from *T. turnerae* extracts eluted at retention times of 12.55, 13.5, 11.3, and 15 min, respectively. Compound 5 from *G. sunshinyii* extracts eluted at 12 min. Due to incomplete resolution of acyl chain isomers during the initial collections, fractions were subjected to a repeated round of HPLC purification using the same gradient to improve purity.

### Antimicrobial assay

Frozen bacterial stocks were revived by streaking onto appropriate media: *E. coli* C600 (gift from M. Mulvey, University of Utah) and BW25113 (BioResource Project-*E. coli*, National Institute of Genetics, Japan) on Luria-Bertani (LB) agar; Δ*mlaA E. coli* BW25113 mutant (BioResource Project-*E. coli*, National Institute of Genetics, Japan) on LB agar supplemented with 10 µg mL^−1^ kanamycin; and *Acinetobacter baumannii* ATCC 19606 on Mueller–Hinton (MH) agar. Plates were then incubated at 37 °C for 8 to 12 hours. Isolated colonies were then inoculated into corresponding liquid broths and cultured with agitation at 37 °C, 150 rpm for 6 to 8 hours. The resulting cultures were standardized to a 0.5 McFarland turbidity (approximately 1 × 10^8^ cells mL^−1^) and diluted 1 : 200 to create the working inoculum. Then, 200 µL of this bacterial suspension was added to 96-well microtiter plates alongside test compounds in an eight-point concentration series, prepared by two-fold serial dilution starting at 32 µg mL^−1^. After 18–20 hours of incubation at 37 °C, 150 rpm, 20 µL of an MTT solution (5 mg mL^−1^) was dispensed into each well and incubated for 2 hours. The precipitated formazan crystals were then solubilized in 100 µL of DMSO for 1 hour, after which the final optical density was measured at 570 nm using a Biotek Synergy 2 microplate reader.

### Creation of Fig. 1A

The shipworm was first sketched by an author and then converted to a cartoon by ChatGPT, with oversight from authors. Other elements were created using BioRender.

## Conflicts of interest

B. W. M. and E. W. S. are co-inventors on a patent application concerning turnercyclamycins.

## Supplementary Material

CB-OLF-D6CB00220J-s001

## Data Availability

The data supporting this article have been included as part of the supplementary information (SI). Supplementary information is available. See DOI: https://doi.org/10.1039/d6cb00220j.
