# Supplementary material for: Lipid interactions couple turnercyclamycin antibacterial activity with carbohydrate-active enzyme packaging in membrane vesicles
Source: RSC Chem Biol. 2026 Oct 9. Online ahead of print. doi: 10.1039/d6cb00220j (PMC13682526; doi:10.1039/d6cb00220j)
Supplement: Supplementary file 1 [file CB-OLF-D6CB00220J-s001.pdf]

This journal is © The Royal Society of Chemistry

Supplemental Information

Lipid interactions couple turnercyclamycin antibacterial activity with carbohydrate-active enzyme packaging in membrane vesicles

Bailey W. Miller,[a*] Albebson L. Lim,[a*] Mark T. Gasser,[b] Zhenjian Lin,[a] Daniel L. Distel,[c#] Eric W. Schmidt[a#]

[a]Department of Medicinal Chemistry, University of Utah, Salt Lake City, UT 84112, USA
[b]Johns Hopkins Applied Physics Laboratory, Laurel, MD, 20723, USA
[c]Ocean Genome Legacy Center, Northeastern University, Nahant, MA, 01908, USA

Table of Contents

Figure S1. Minor turnercyclamycins in whole cell extracts (top) versus MVs (bottom)...3
Figure S2. *Gynuella* and *Teredinibacter tur* clusters and Sanger sequencing of truncated Δ*turH*...4
Figure S3. LC-MS (A, B) and MS/MS (C) of dehydroxy-turnercyclamycin A (**1a**) from Δ*turH*...5
Figure S4. LC-MS (A, B) and MS/MS (C) of dehydroxy-turnercyclamycin B (**2a**) from Δ*turH*...5
Figure S5. ^{1}H NMR spectrum of **5** in DMSO-*d*$_6$ (500 MHz)...6
Figure S6. ^{13}C NMR spectrum of **5** in DMSO-*d*$_6$ (125 MHz)...6
Figure S7. COSY NMR spectrum of **5** in DMSO-*d*$_6$ (500 MHz)...7
Figure S8. TOCSY NMR spectrum of **5** in DMSO-*d*$_6$ (500 MHz)...7
Figure S9. HSQC NMR spectrum of **5** in DMSO-*d*$_6$ (500 MHz)...8
Figure S10. HMBC NMR spectrum of **5** in DMSO-*d*$_6$ (500 MHz)...8
Figure S11. ROESY NMR spectrum of **5** in DMSO-*d*$_6$ (500 MHz)...9
Figure S12. MS spectra of minor *G. sunshinyii* turnercyclamycin analogs...9
Figure S13. Maximum likelihood phylogenetic tree of oxygenases...10
Figure S14. Mass spectrometric analysis of turnercyclamycin C (**3**)...11
Figure S15. ^{1}H NMR of turnercyclamycin C (**3**) in CD_3OD (500 MHz)...12
Figure S16. COSY NMR of turnercyclamycin C (**3**) in CD_3OD (500 MHz)...12
Figure S17. HSQC NMR of turnercyclamycin C (**3**) in CD_3OD (500 MHz)...13
Figure S18. HMBC NMR of turnercyclamycin C (**3**) in CD_3OD (500 MHz)...13
Figure S19. Mass spectrometric analysis of turnercyclamycin E (**4**)...14
Figure S20. ^{1}H NMR of turnercyclamycin E (**4**) in DMSO-*d*$_6$ (500 MHz)...15
Figure S21. Mass spectrometric analysis of turnercyclamycin A1 (**5**)...15
Figure S22. HR-LCMS (A) and MS/MS (B) spectra of turnercyclamycin A butyl ester (**1b**)...16
Figure S23. ^{1}H NMR spectrum of turnercyclamycin A butyl ester (**1b**) in DMSO-*d*$_6$ (500 MHz)...16
Figure S24. COSY NMR spectrum of turnercyclamycin A butyl ester (**1b**) in DMSO-*d*$_6$ (500 MHz)...17
Figure S25. HSQC NMR spectrum of turnercyclamycin A butyl ester (**1b**) in DMSO-*d*$_6$ (500 MHz)...18
Figure S26. TOCSY NMR spectrum of turnercyclamycin A butyl ester (**1b**) in DMSO-*d*$_6$ (500 MHz)...18
Figure S27. Raw results from antimicrobial assays...19

Figure S28. Butyl ester activity against bacteria...20
Figure S29. Light microscopy of *E. coli* cells treated under different conditions...20
Table S1. Primers used in this study...21
Table S2. NMR chemical shift assignments compared to literature values for **2**...21

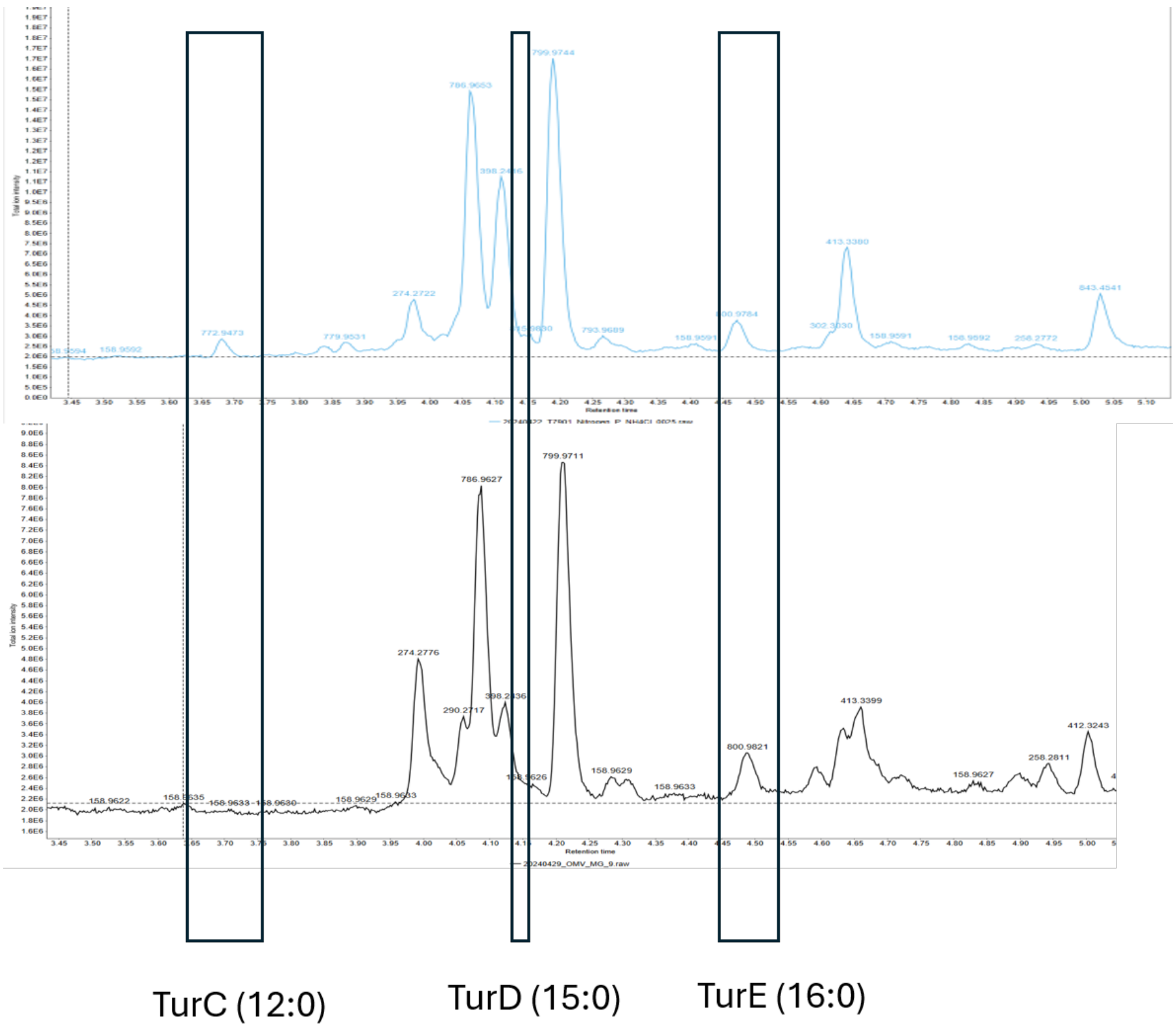

Figure S1. Minor turnercyclamycins in whole cell extracts (top) versus MVs (bottom).

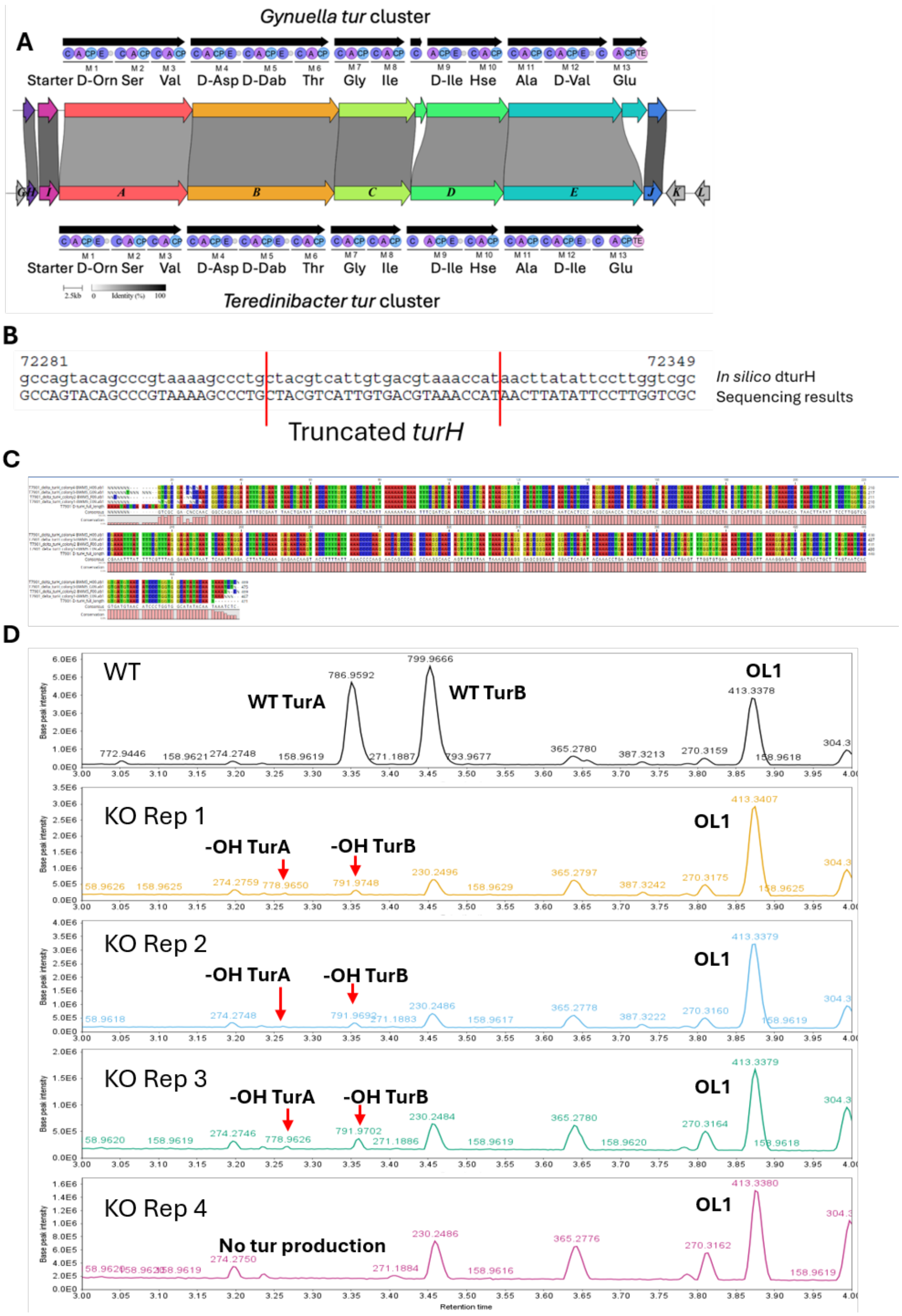

Figure S2. (A) Comparison of *Gynuella* and *Teredinibacter tur* clusters. They are very similar except in the oxidase region. (B) Sanger sequencing of truncated Δ*turH* gene after allelic exchange. (C) Sanger sequencing results of the junction region were identical for 4 replicates of the Δ*turH* strain. (D) Three of the four replicates displayed the dehydroxylated turnercyclamycin chemotype. The fourth replicate did not produce turnercyclamycins.

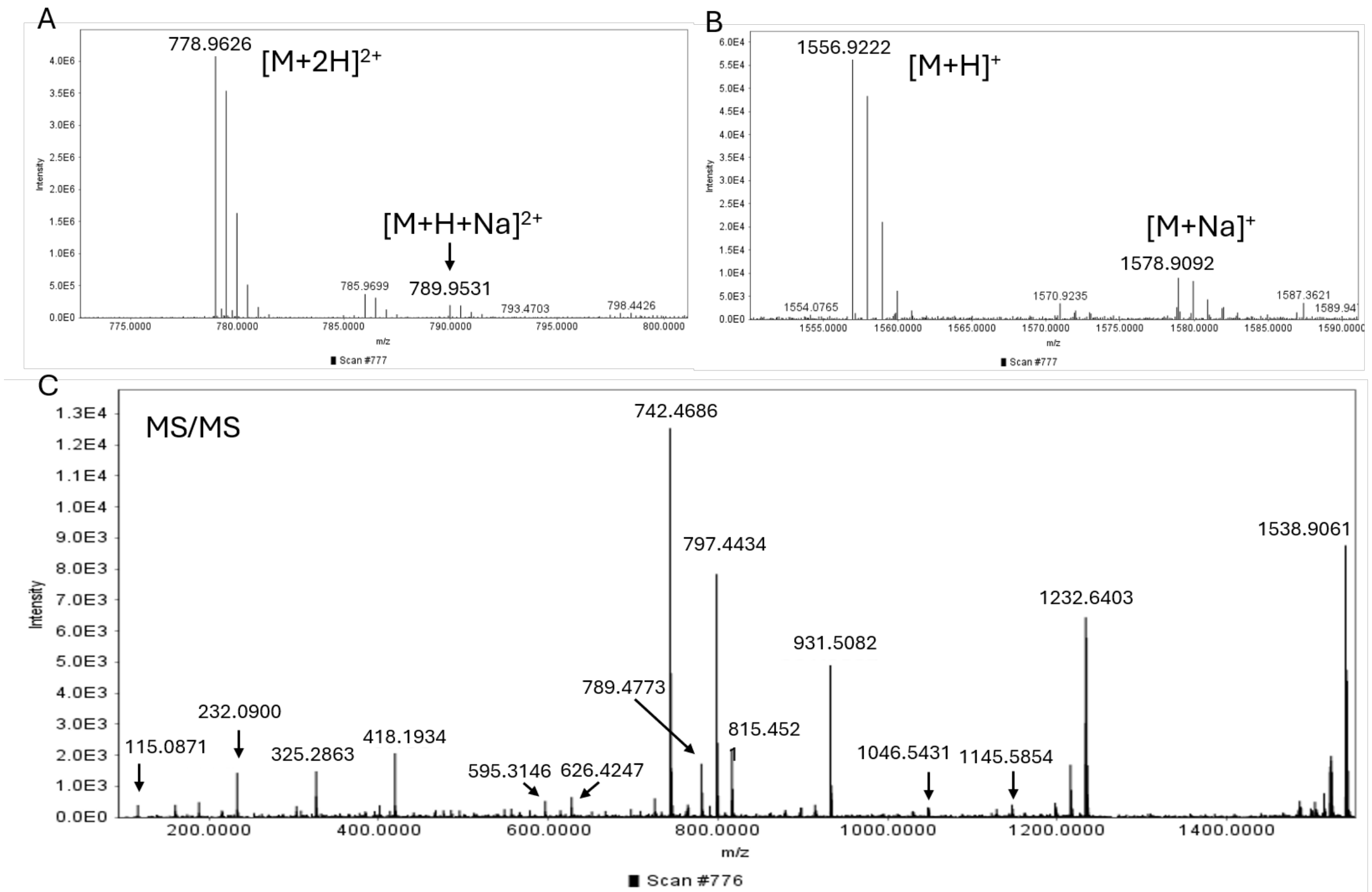

Figure S3. LC-MS (A, B) and MS/MS (C) of dehydroxy-turnercyclamycin A (**1a**) from Δ*turH*.

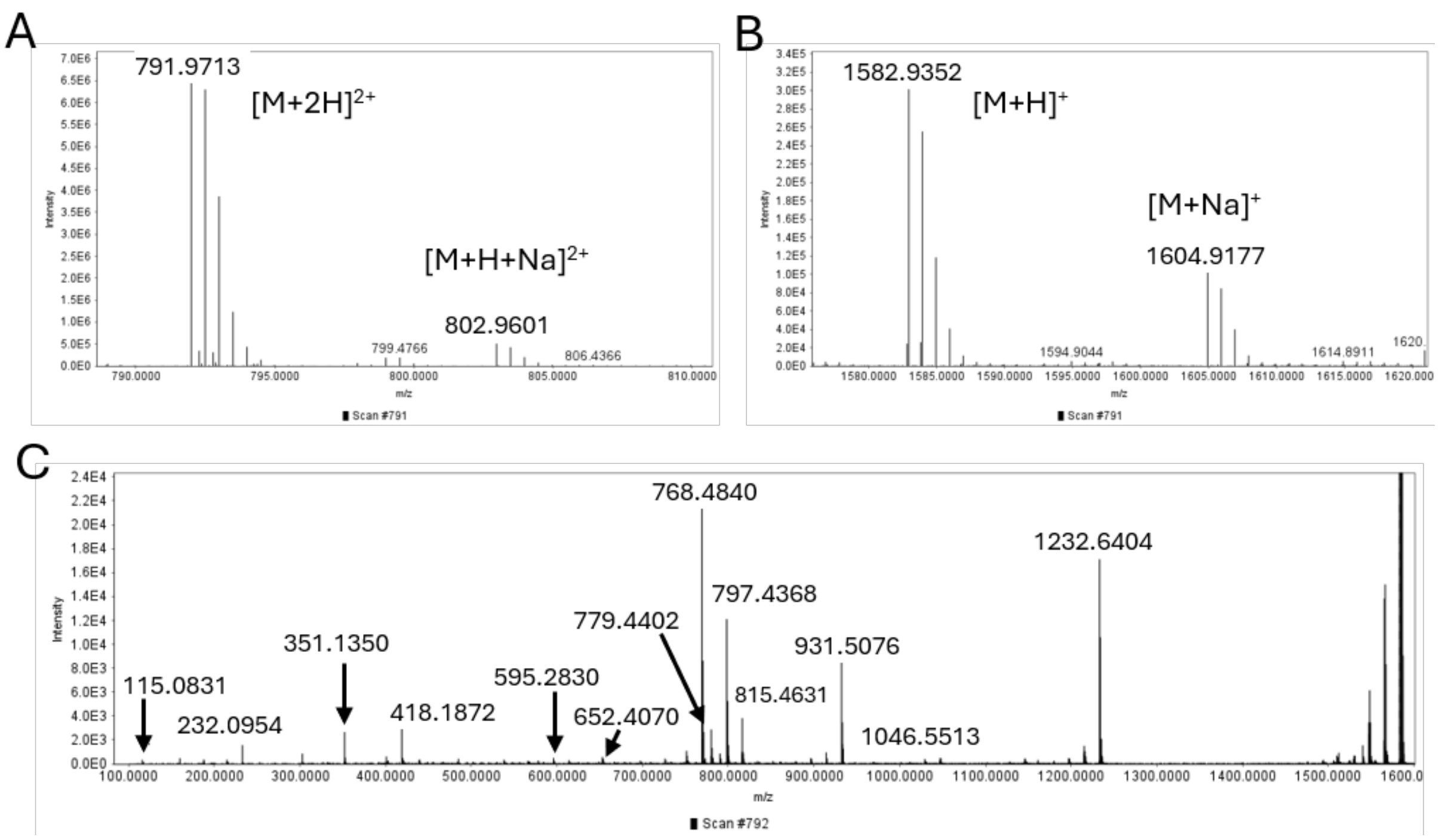

Figure S4. LC-MS (A, B) and MS/MS (C) of dehydroxy-turnercyclamycin B (**2a**) from Δ*turH*.

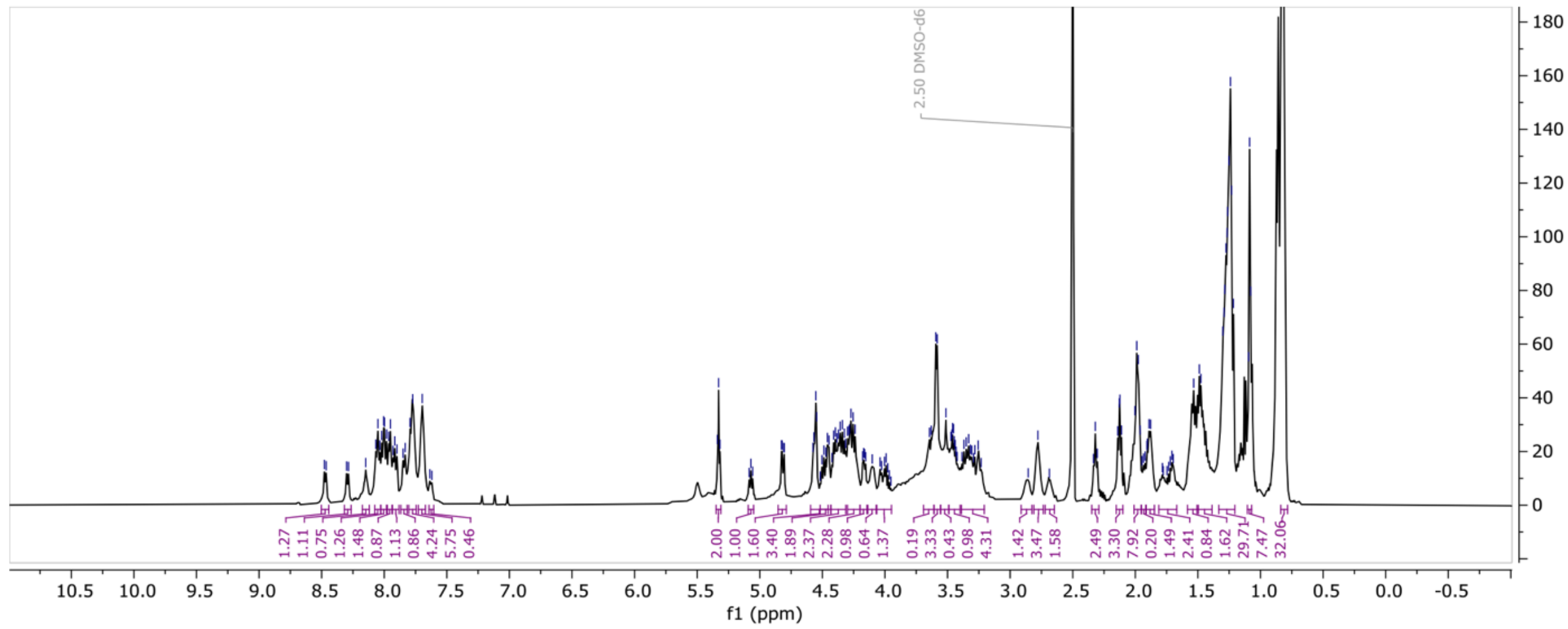

Figure S5. ^{1}H NMR spectrum of **5** in DMSO-d_6 (500 MHz).

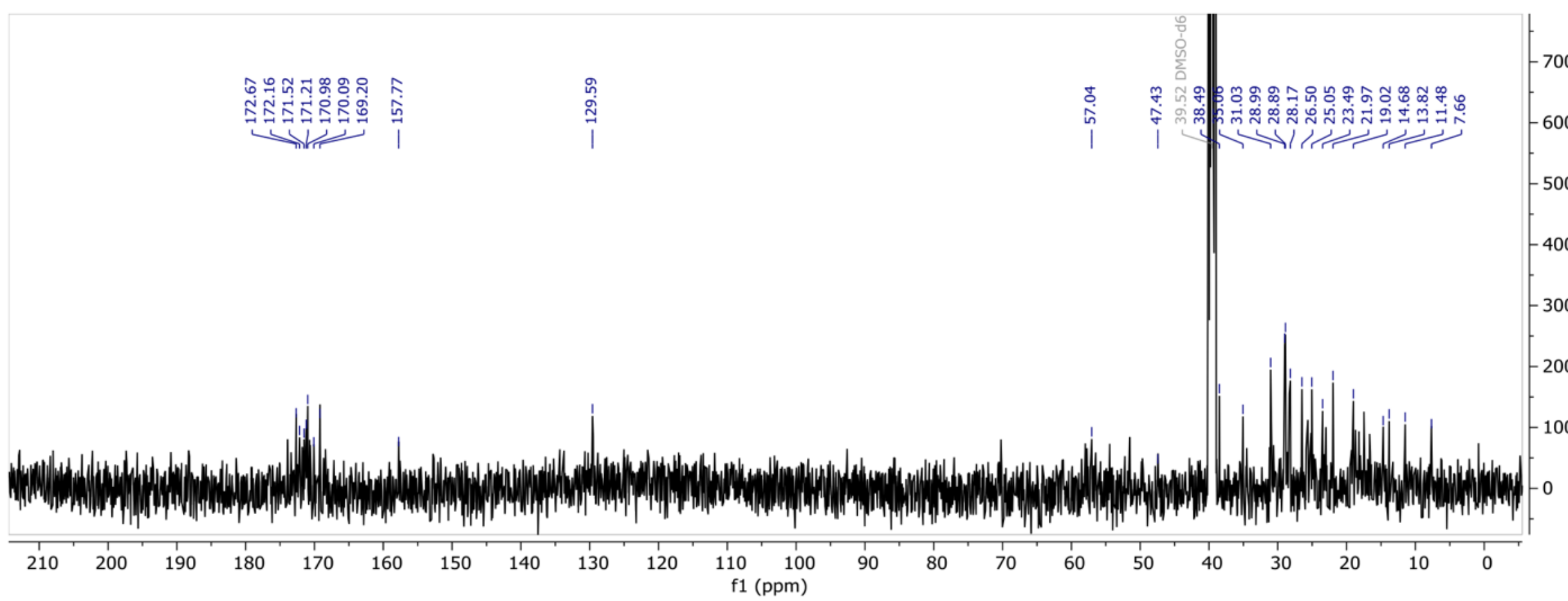

Figure S6. ^{13}C NMR spectrum of **5** in DMSO-d_6 (125 MHz).

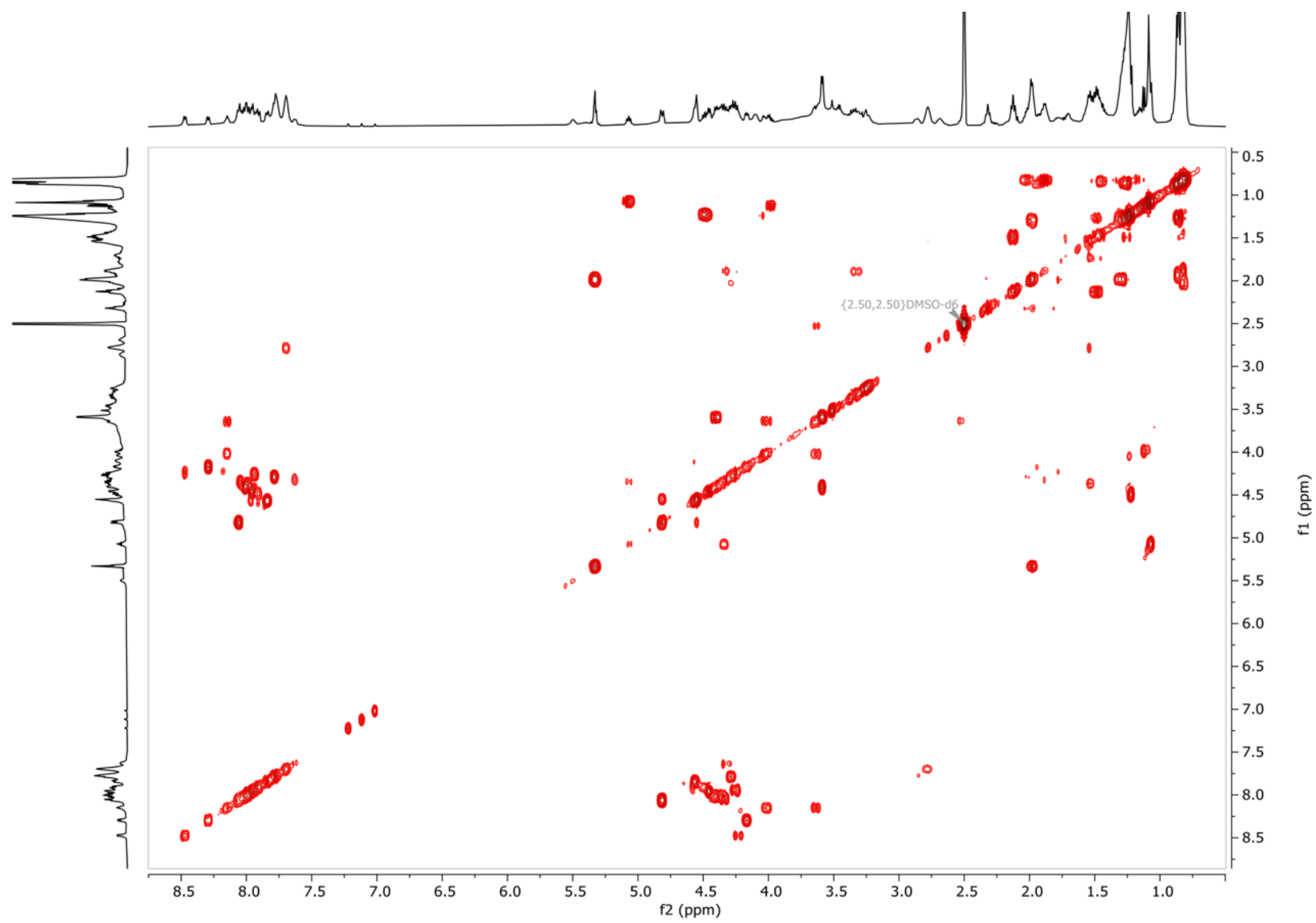

Figure S7. COSY NMR spectrum of **5** in DMSO-d_6 (500 MHz).

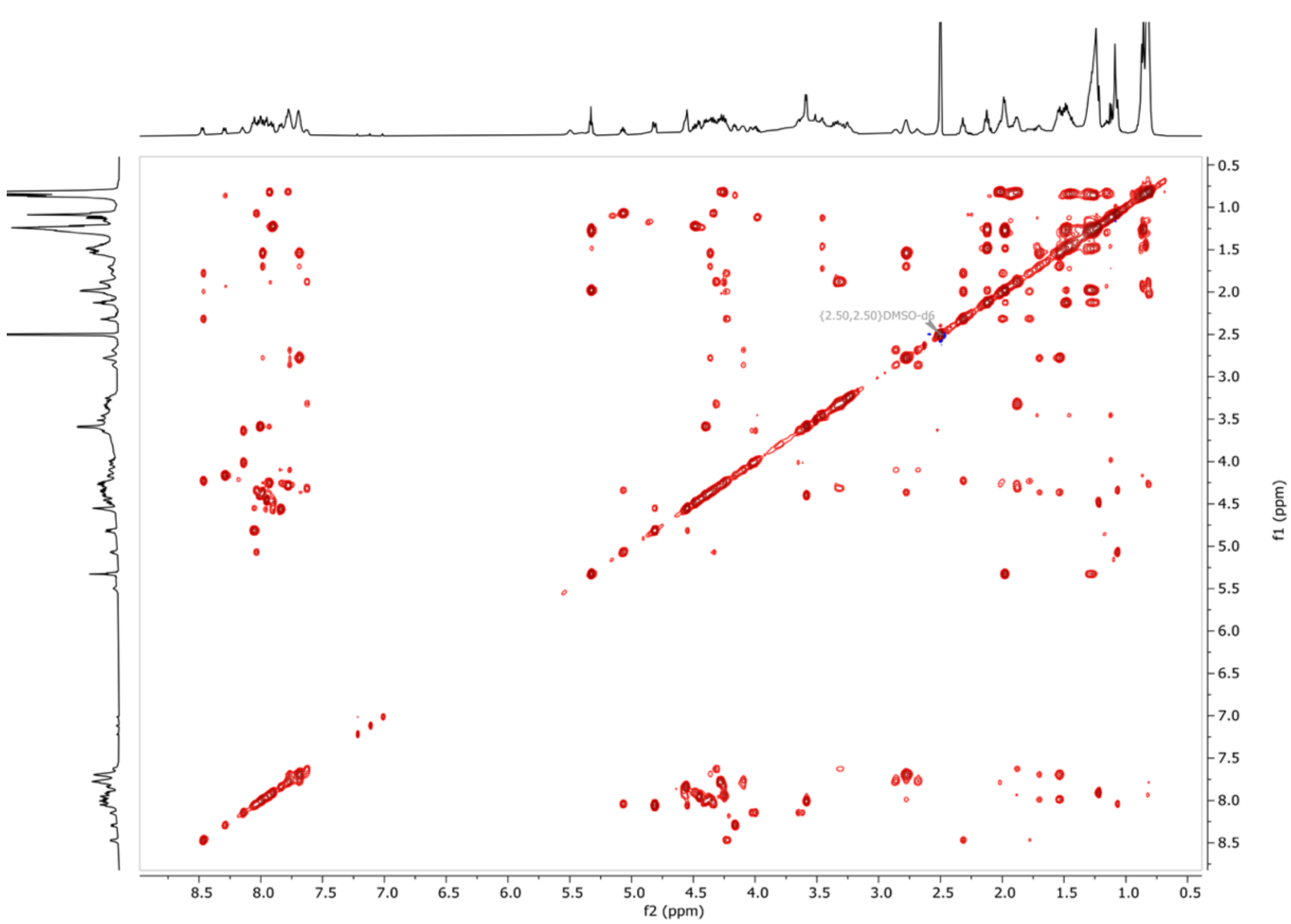

Figure S8. TOCSY NMR spectrum of **5** in DMSO-d_6 (500 MHz).

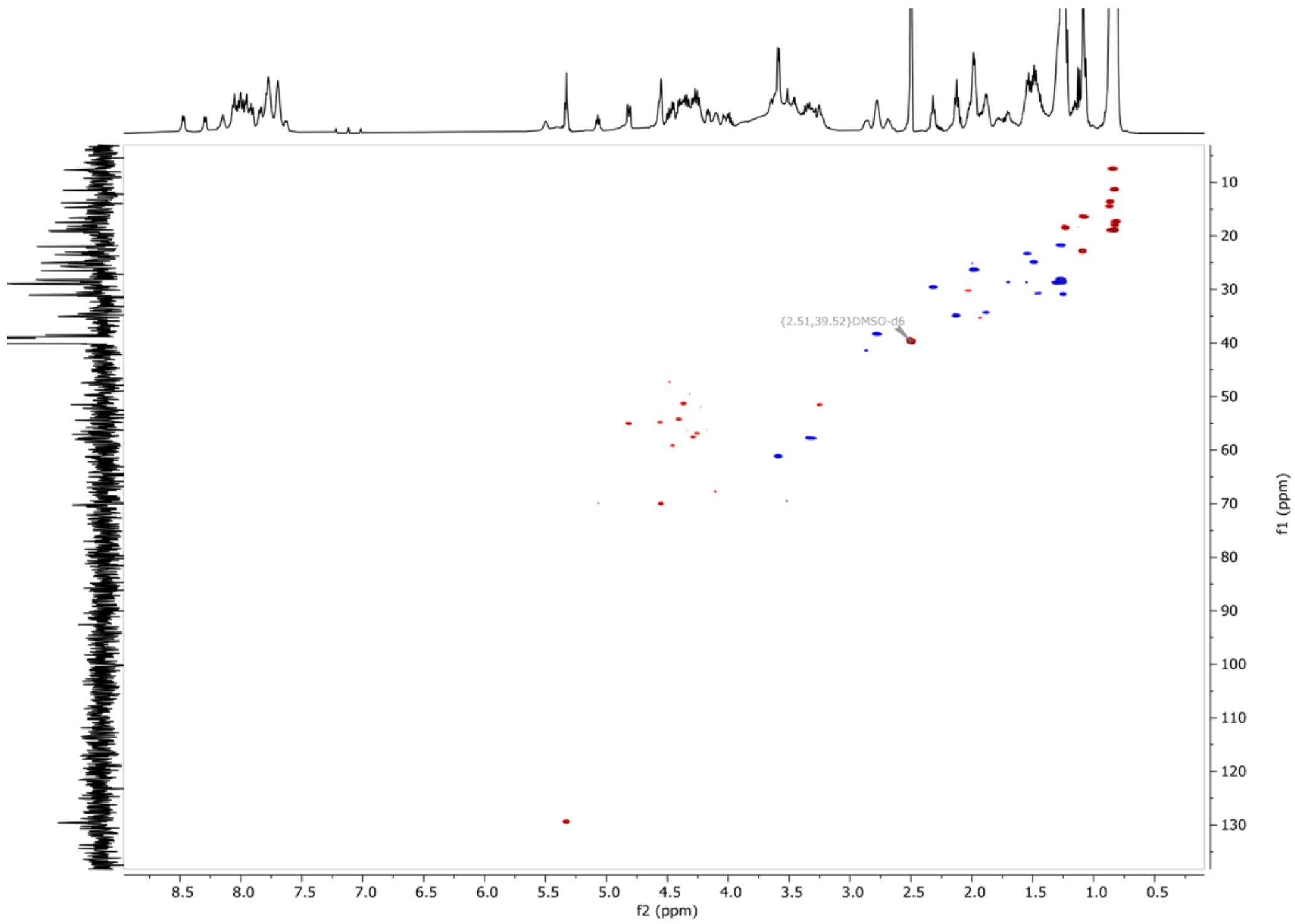

Figure S9. HSQC NMR spectrum of **5** in DMSO-d_6 (500 MHz).

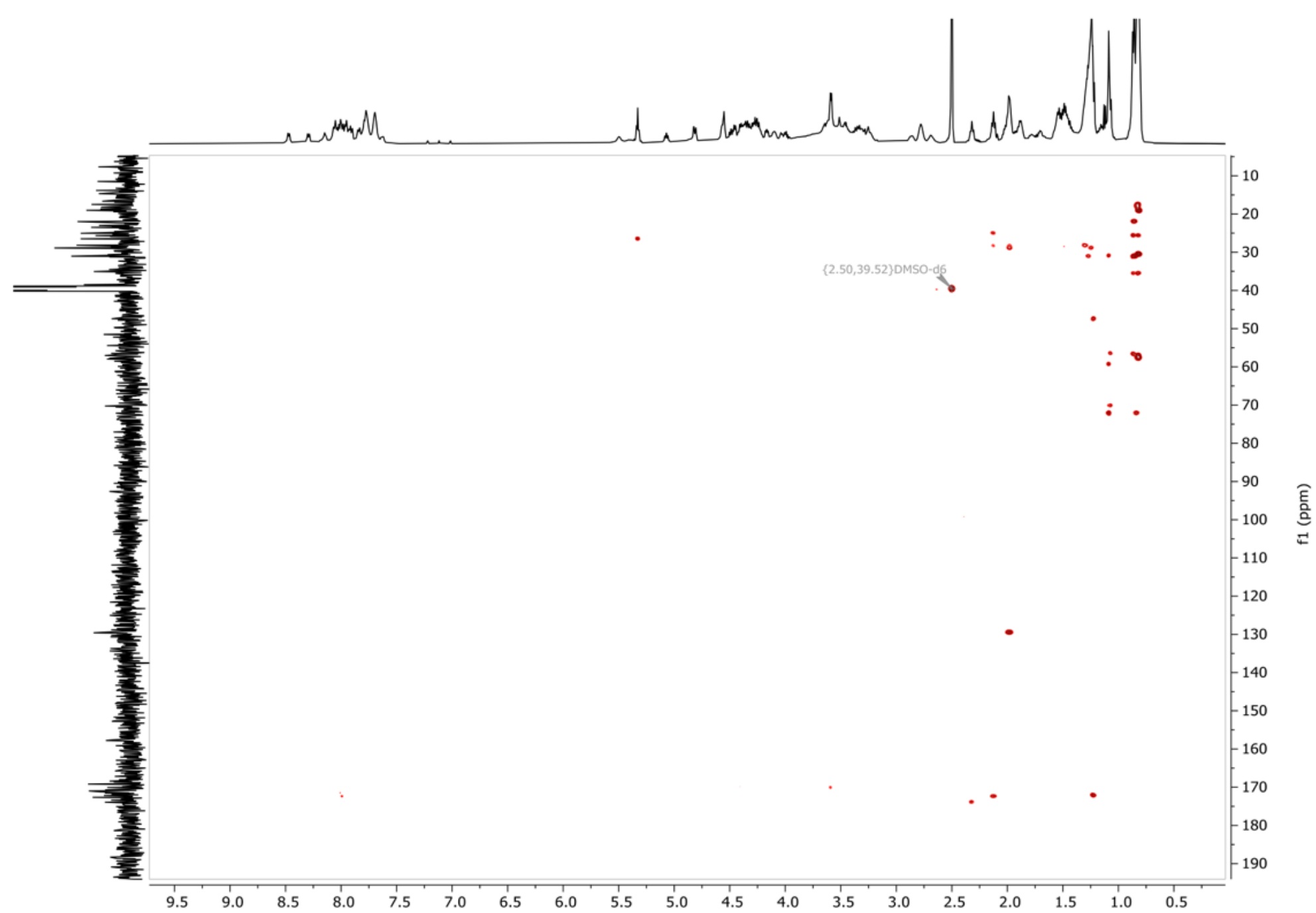

Figure S10. HMBC NMR spectrum of **5** in DMSO-d_6 (500 MHz).

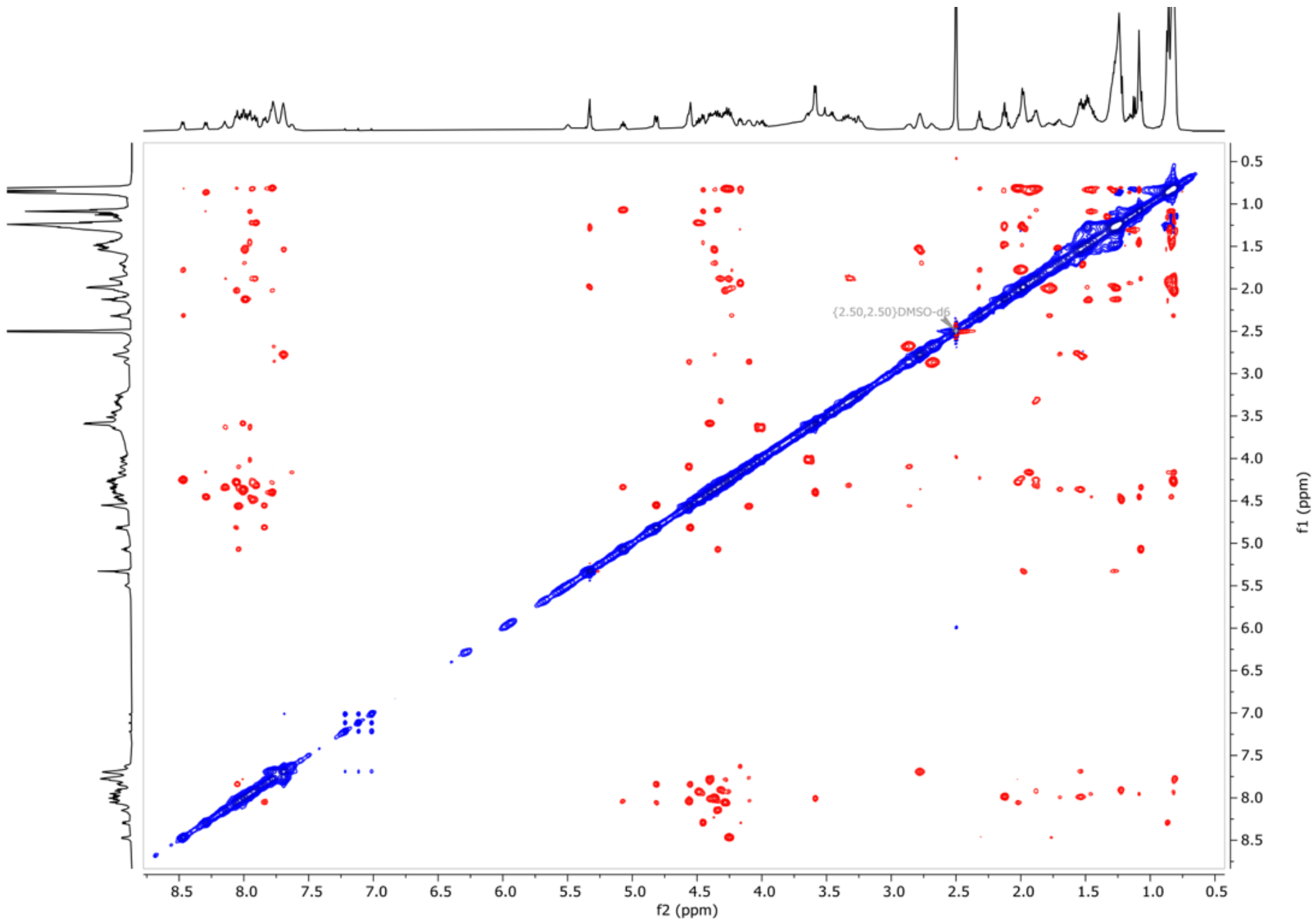

Figure S11. ROESY NMR spectrum of **5** in DMSO-d_6 (500 MHz).

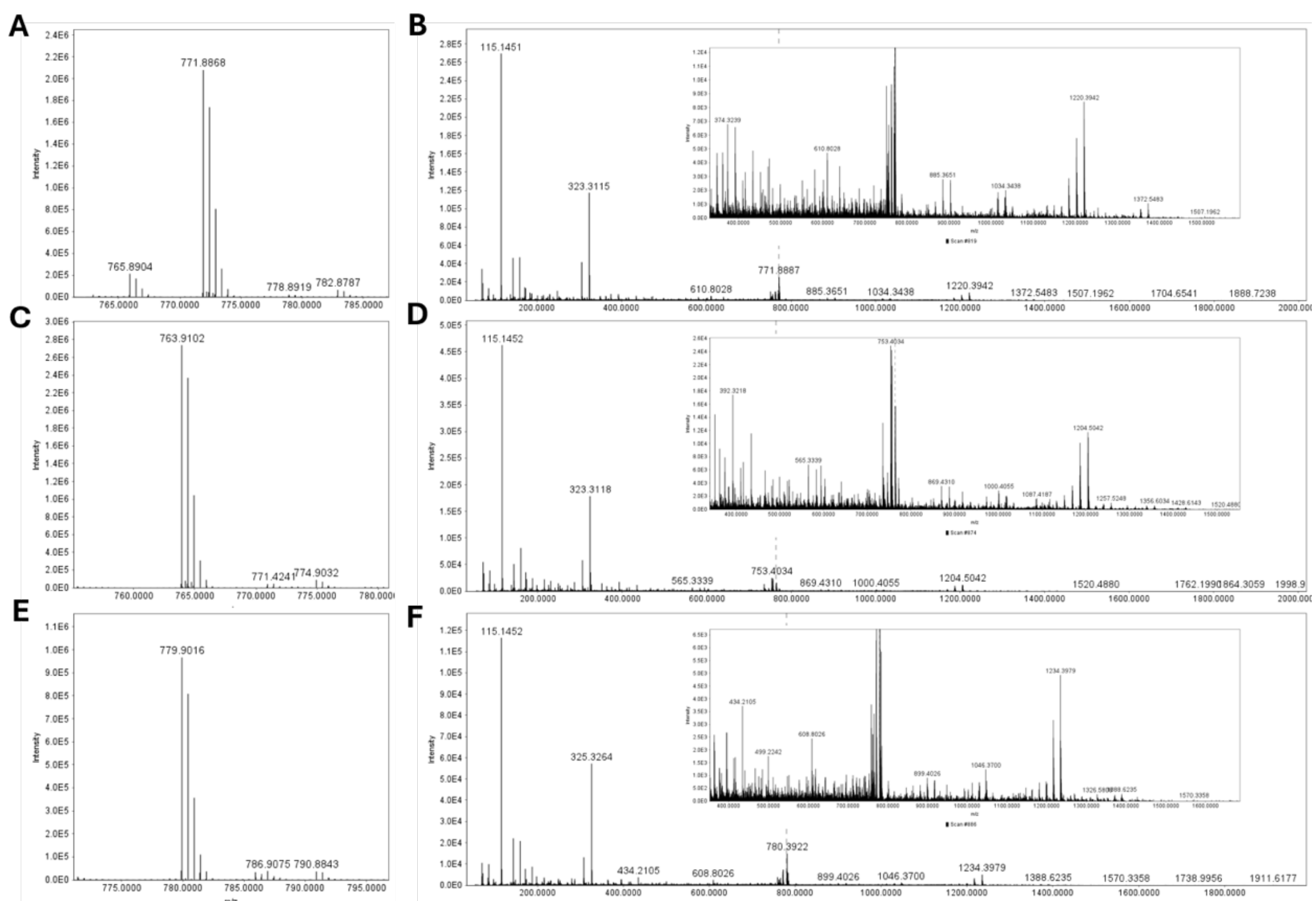

Figure S12. LC-MS spectra (A, C, E) and LC-MS/MS fragmentation spectra (B, D, F) of minor *G. sunshinyii* turnercyclamycin analogs.

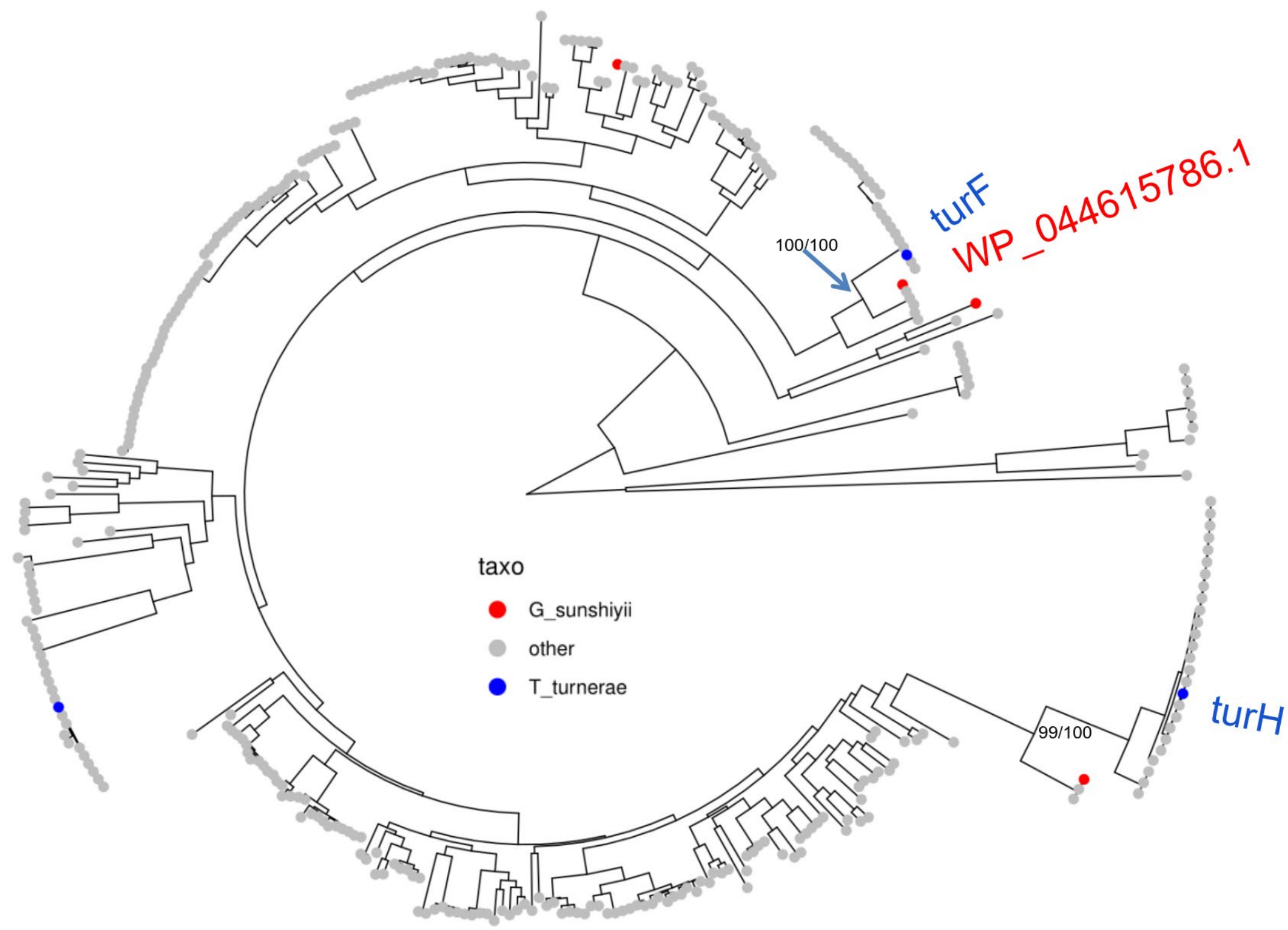

Figure S13. Maximum likelihood phylogenetic tree of alpha-ketoglutarate-dependent oxygenase protein sequences. Nodes were supported by the Shimodaira-Hasegawa likelihood ratio test and ultrafast bootstrap.

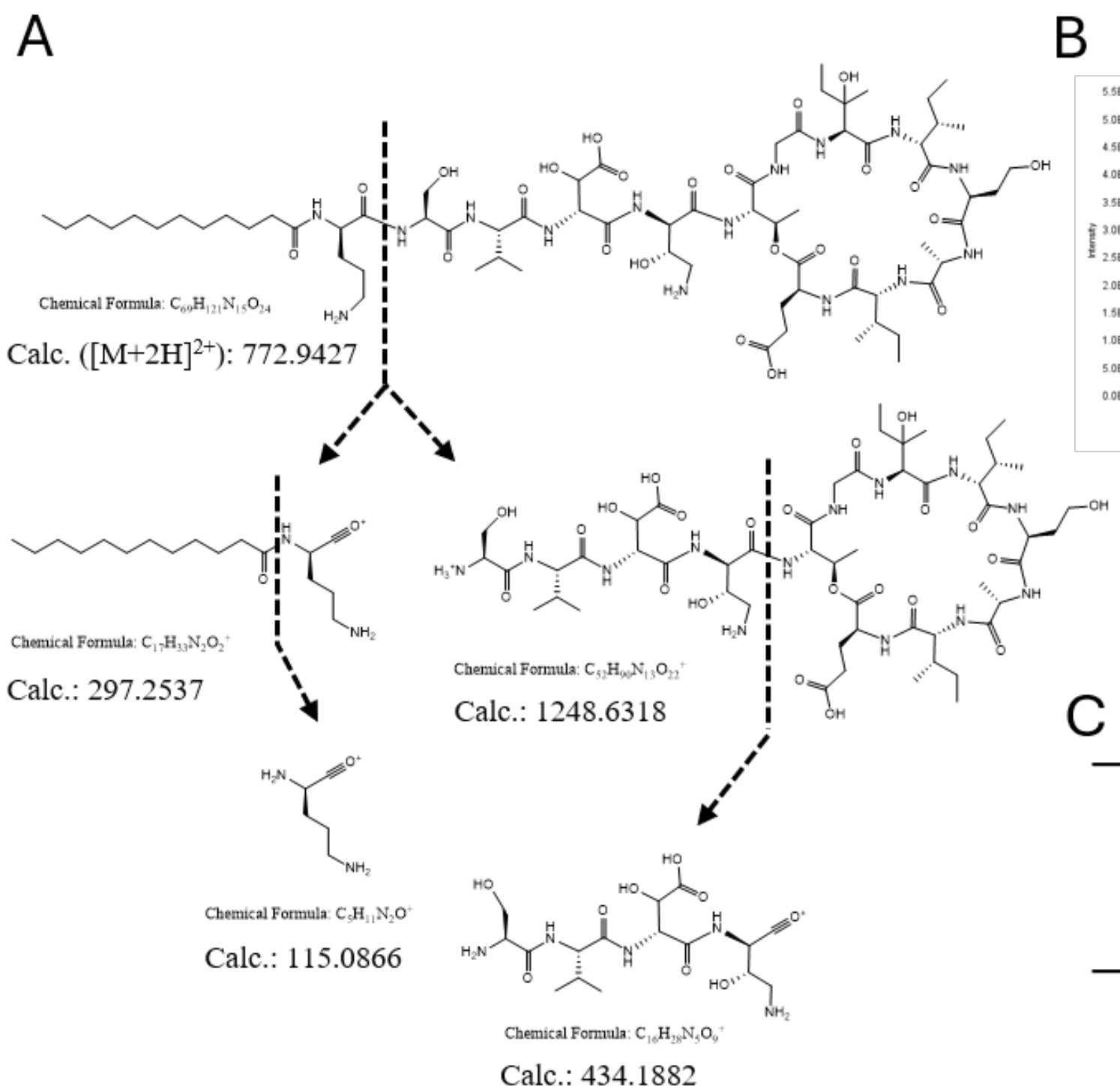

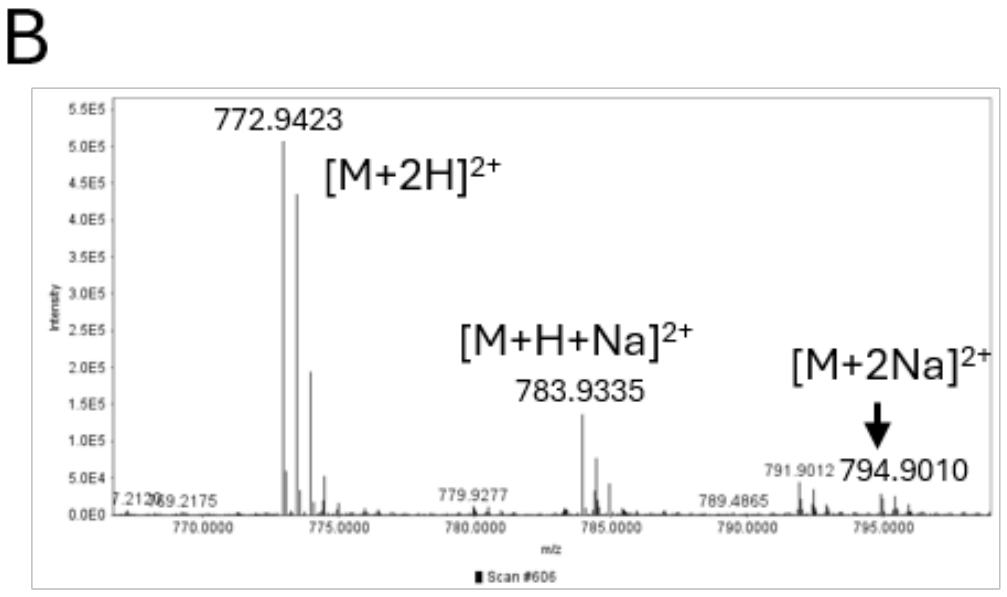

C

Observed	Calculated	Δ (ppm)
115.0860	115.0866	-5.2
297.2524	297.2537	-4.4
1248.6415	1248.6318	7.8

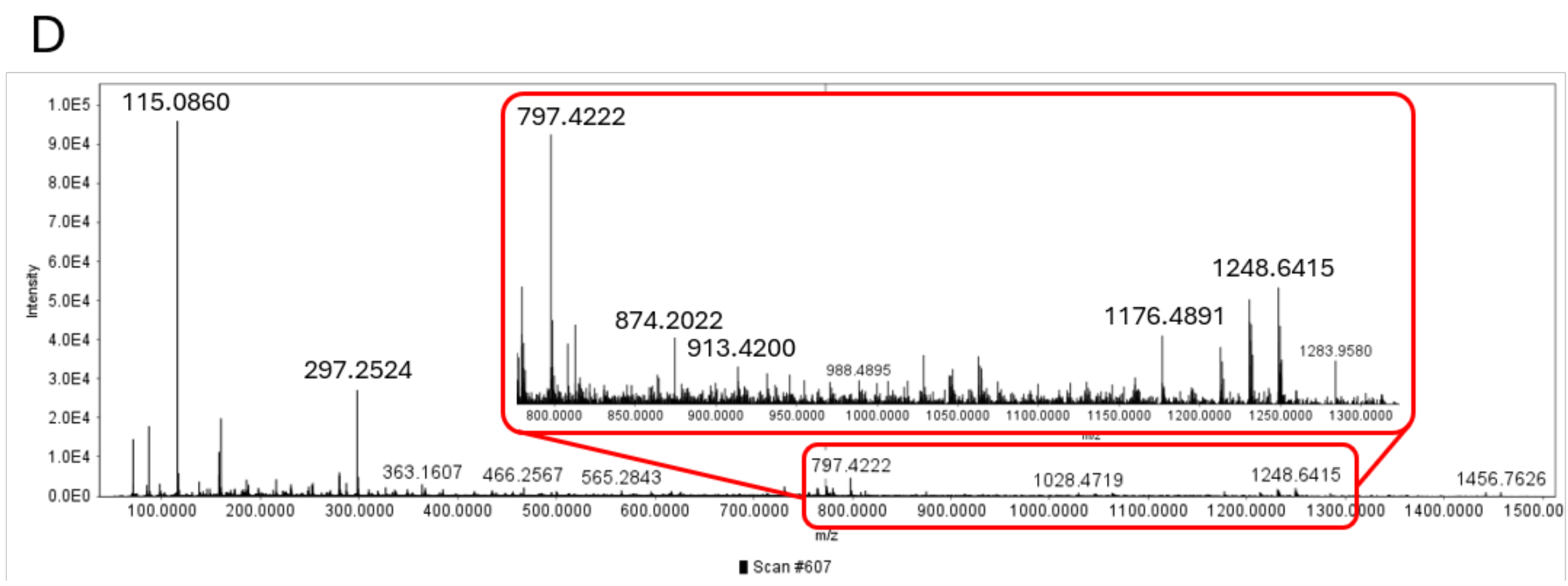

Figure S14. Mass spectrometric analysis of turnercyclamycin C (**3**). (A) Parent molecule and key fragmentation pathway of **3**. (B) LC-ESI-HRqTOFMS of **3**. (C) Observed and calculated *m/z* values for fragment ions generated from **3**. (D) LC-MS/MS spectrum of **3**.

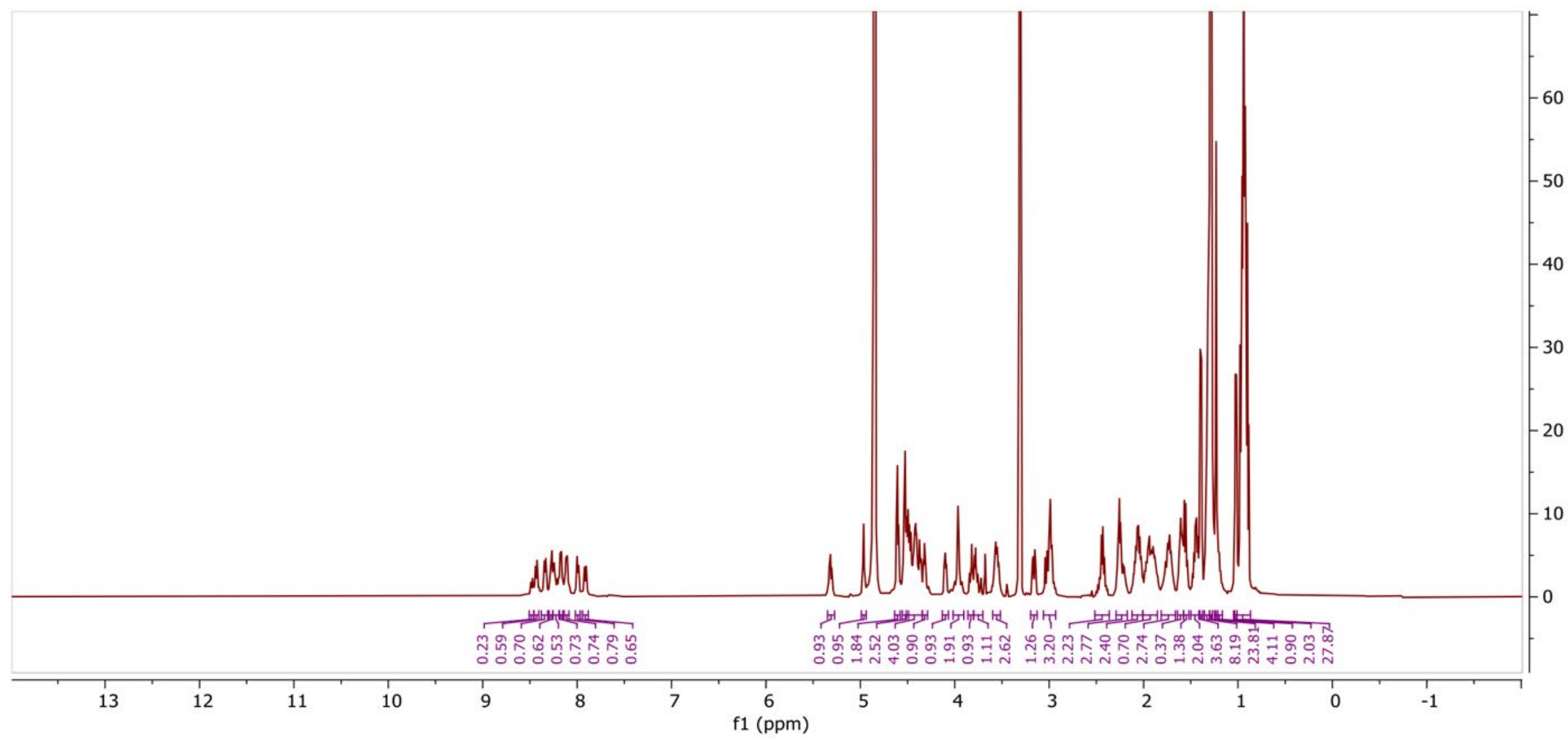

Figure S15. ^{1}H NMR of turnercyclamycin C (**3**) in CD_3OD (500 MHz).

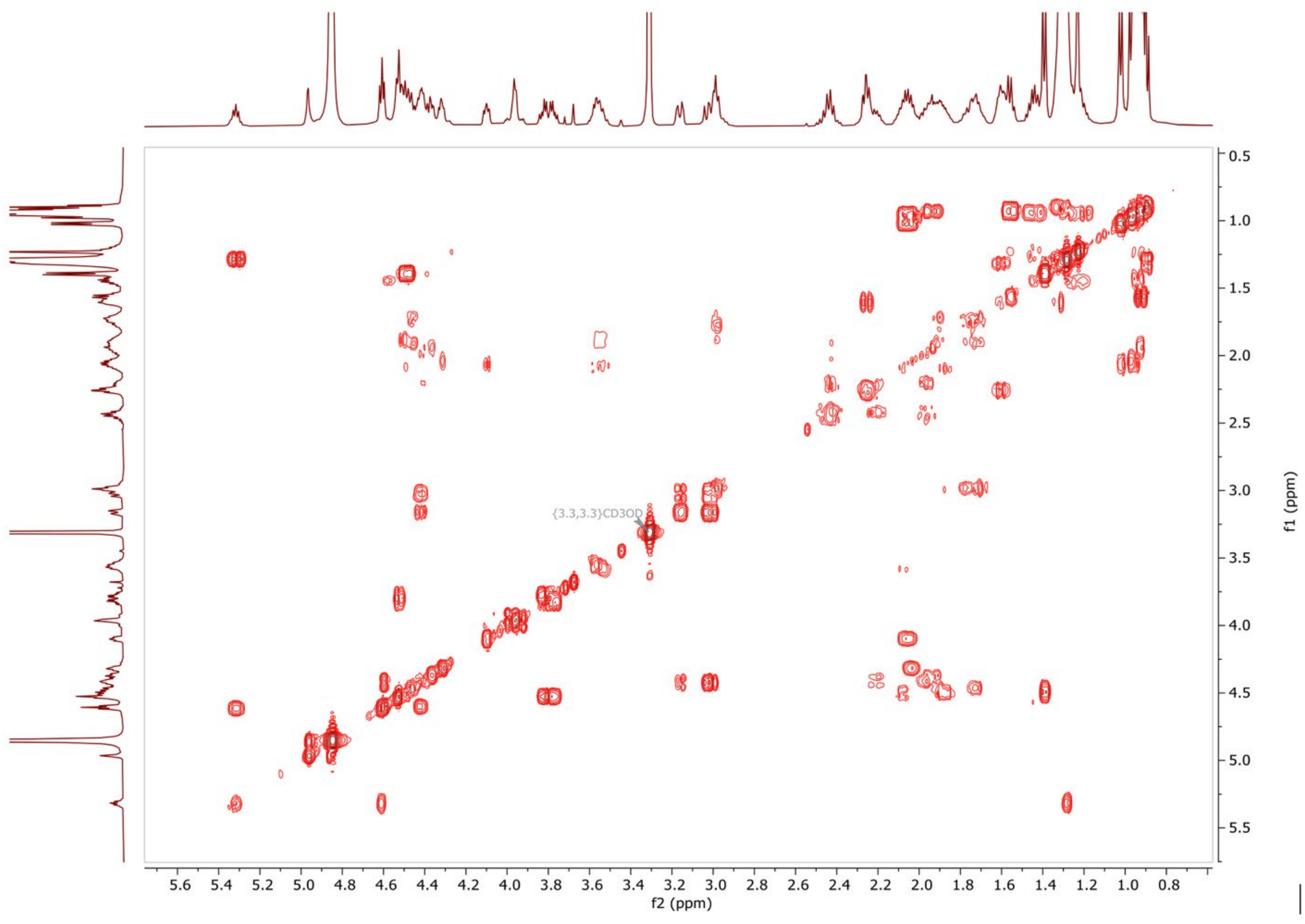

Figure S16. COSY NMR of turnercyclamycin C (**3**) in CD_3OD (500 MHz).

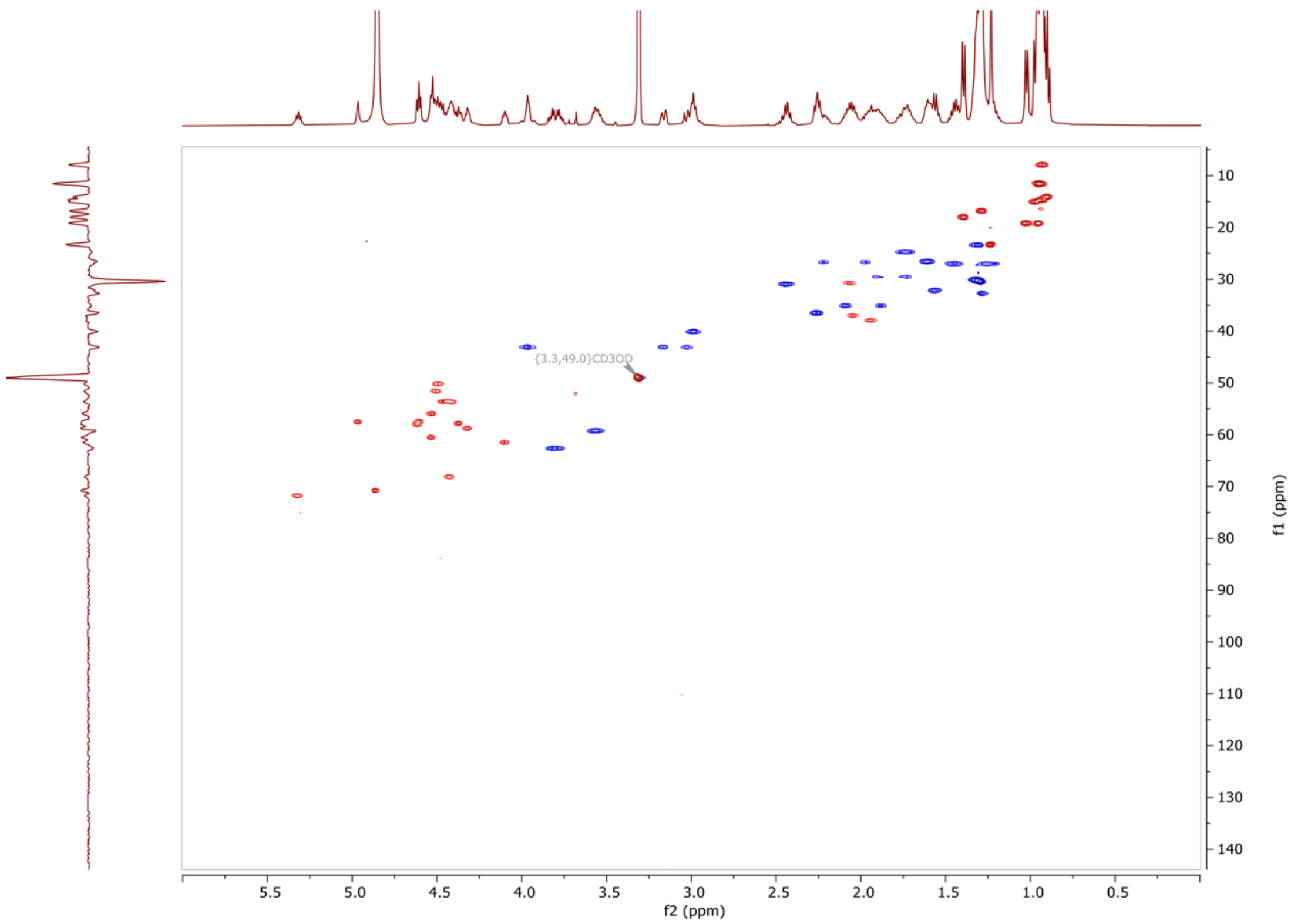

Figure S17. HSQC NMR of turnercyclamycin C (**3**) in CD_3OD (500 MHz).

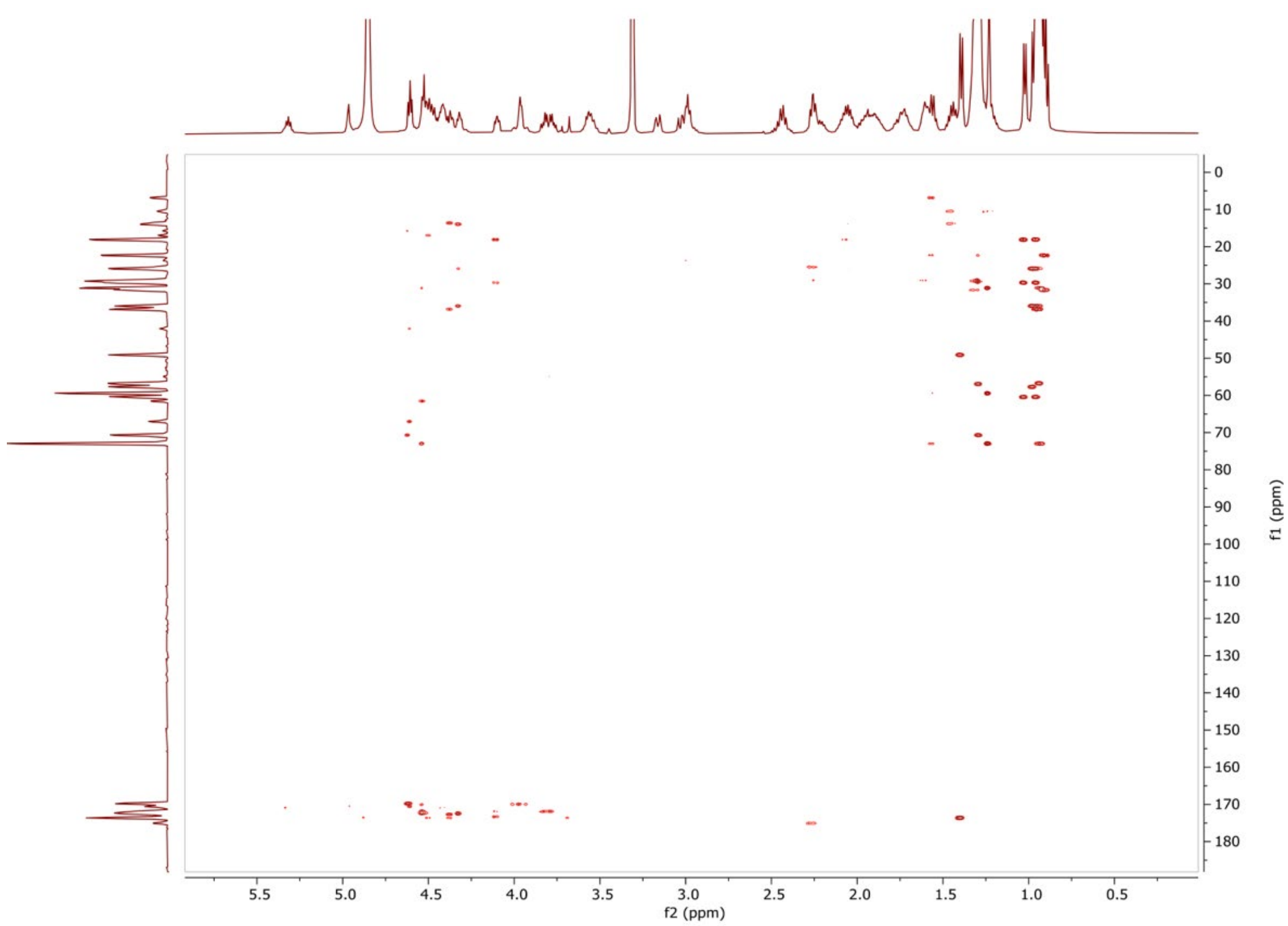

Figure S18. HMBC NMR of turnercyclamycin C (**3**) in CD_3OD (500 MHz).

A

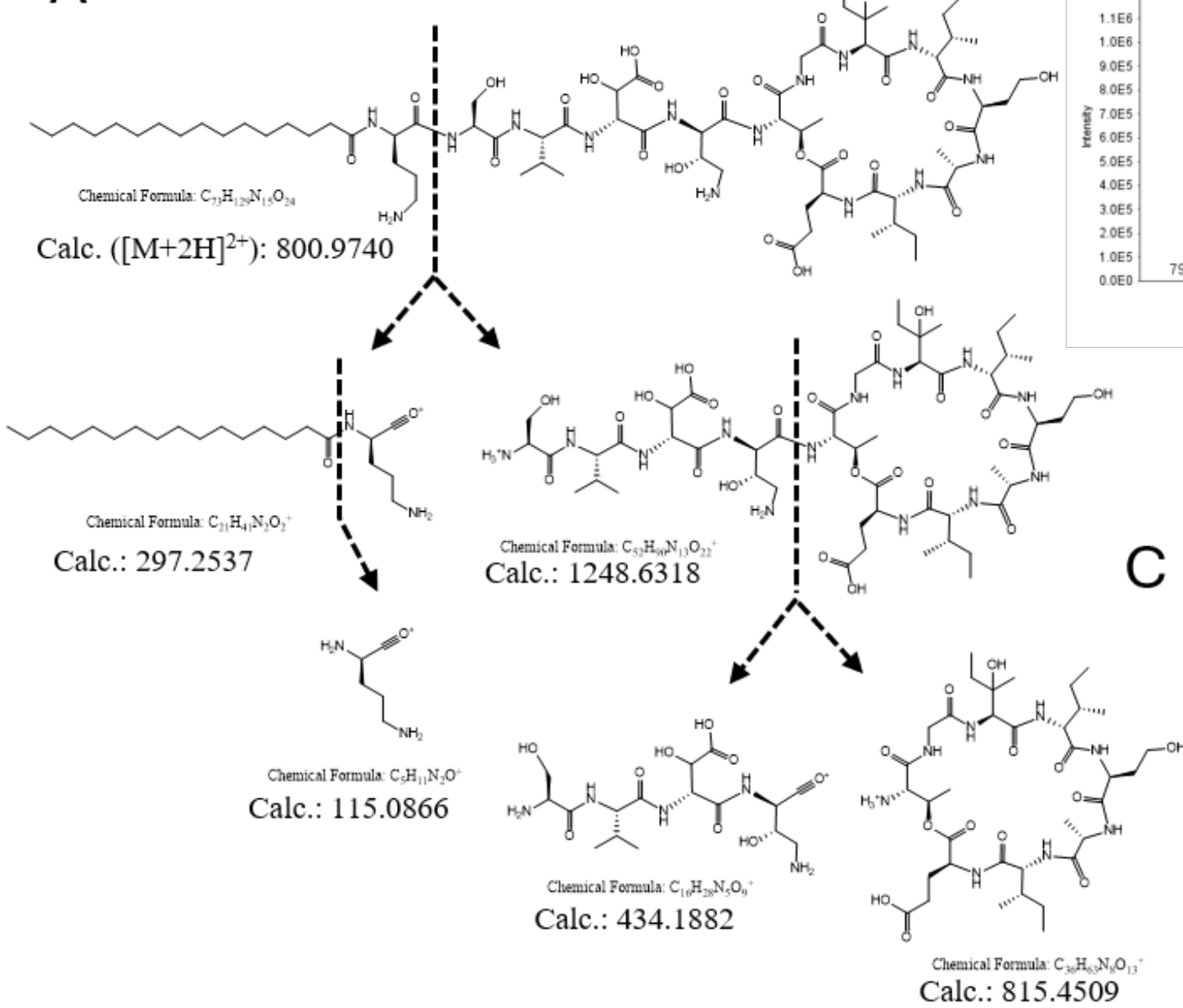

B

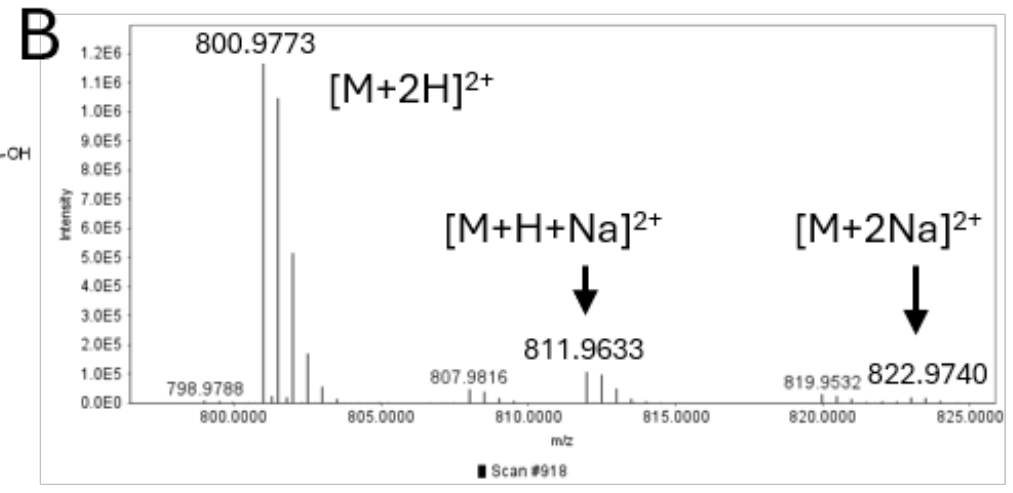

C

Observed	Calculated	Δ (ppm)
115.0858	115.0866	-7.0
353.3151	353.3163	-3.4
815.4429	815.4509	-9.8
1248.6411	1248.6318	7.5

D

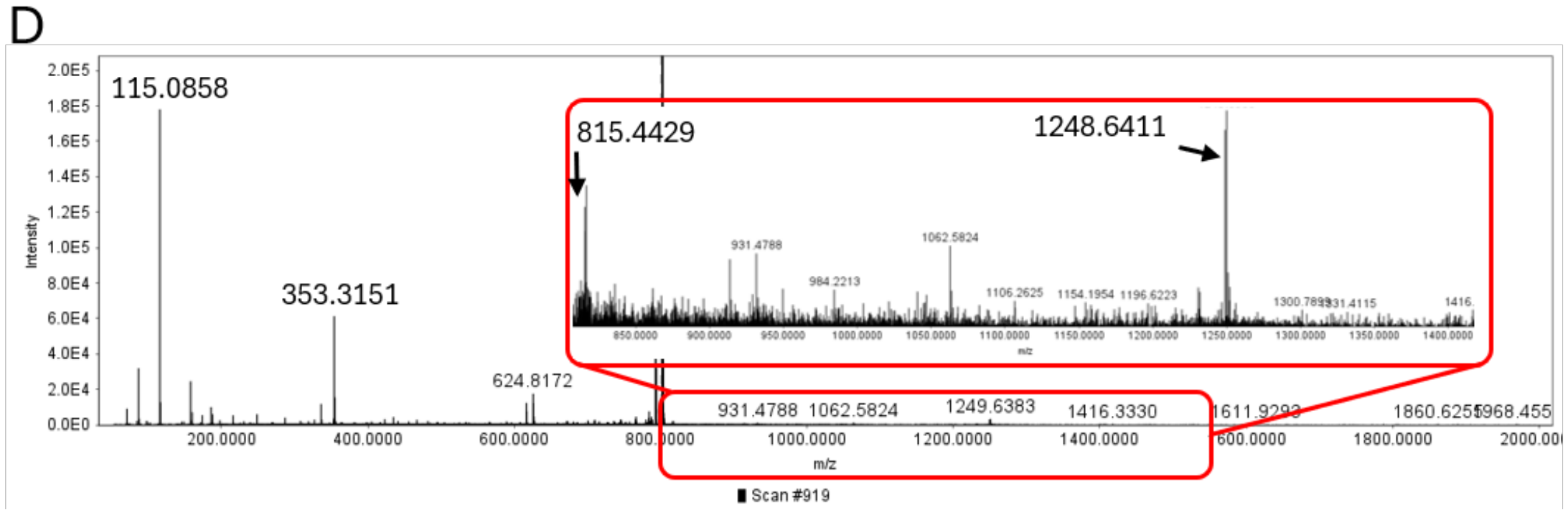

Figure S19. Mass spectrometric analysis of turnercyclamycin E (**4**). (A) Parent molecule and key fragmentation pathway of **4**. (B) LC-ESI-HRqTOFMS of **4**. (C) Observed and calculated *m/z* values for fragment ions generated from **4**. (D) LC-MS/MS spectrum of **4**.

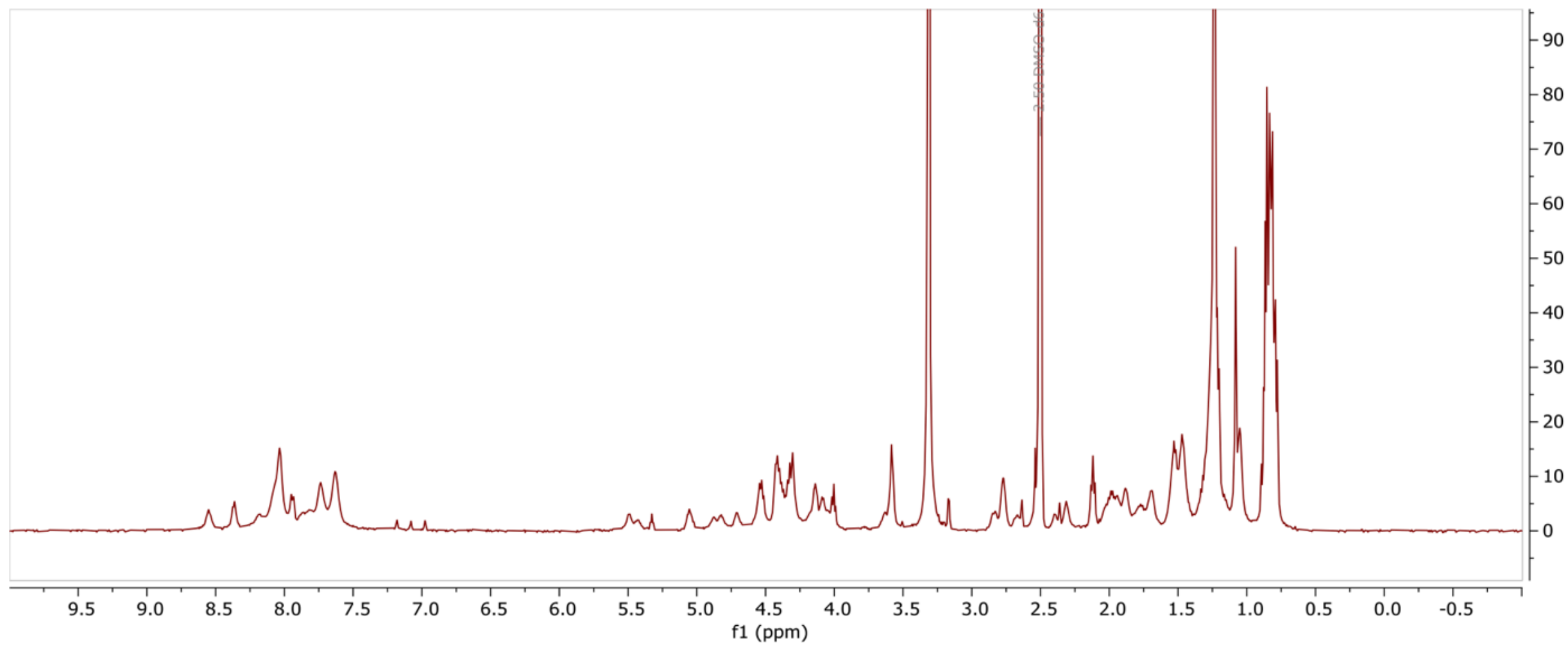

Figure S20. ^{1}H NMR of turnercyclamycin E (**4**) in DMSO-d_6 (500 MHz).

A

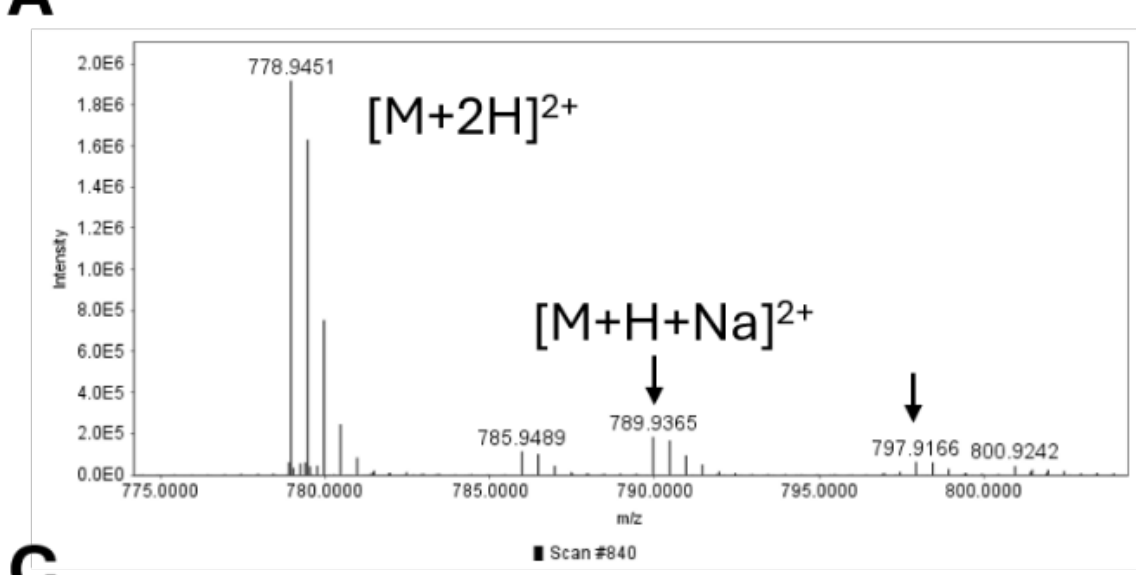

B

Observed	Calculated	Δ (ppm)
115.0867	115.0866	0.9
323.2674	323.2693	-5.9

C

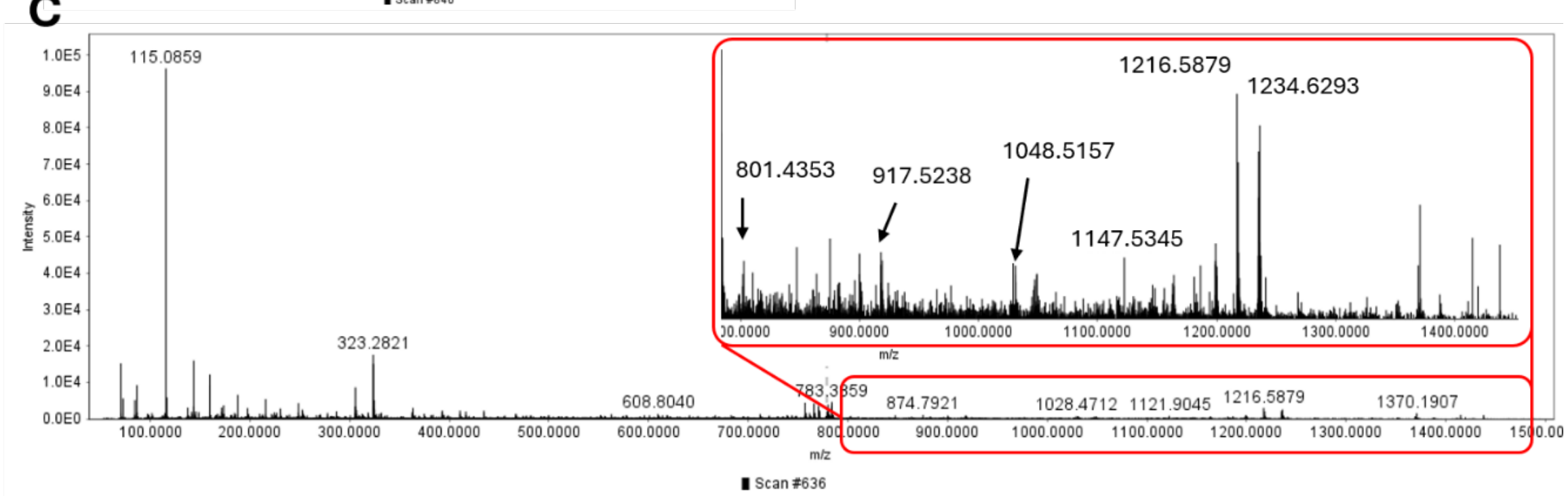

Figure S21. (A) HR-LCMS spectrum, (B) fragment mass analysis, and (C) MS/MS spectrum of turnercyclamycin A1 (**5**).

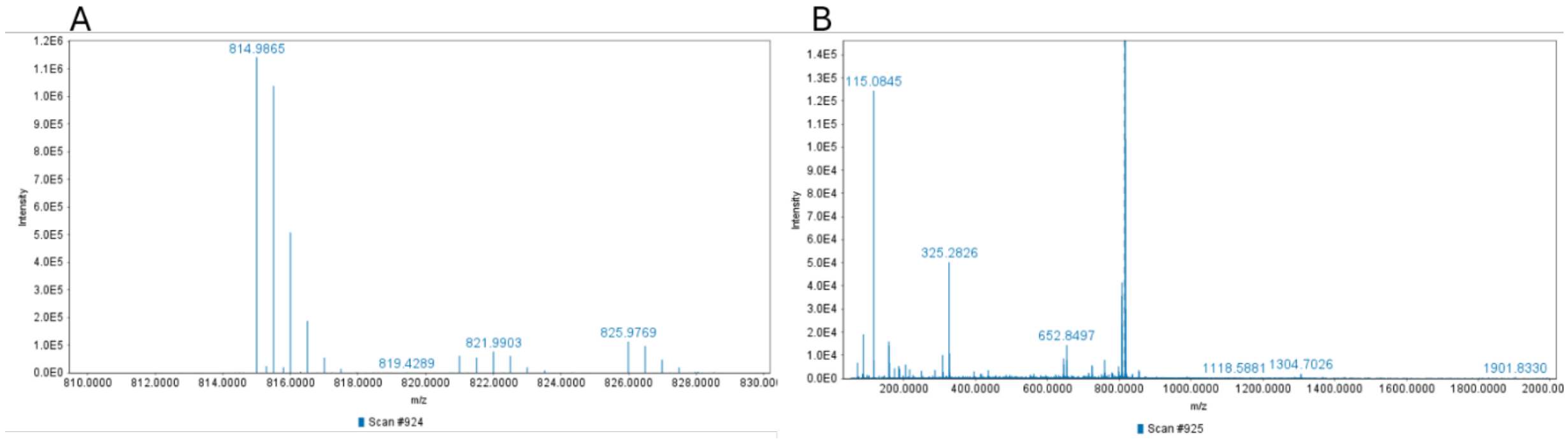

Figure S22. HR-LCMS (A) and MS/MS (B) spectra of turnercyclamycin A butyl ester (**1b**).

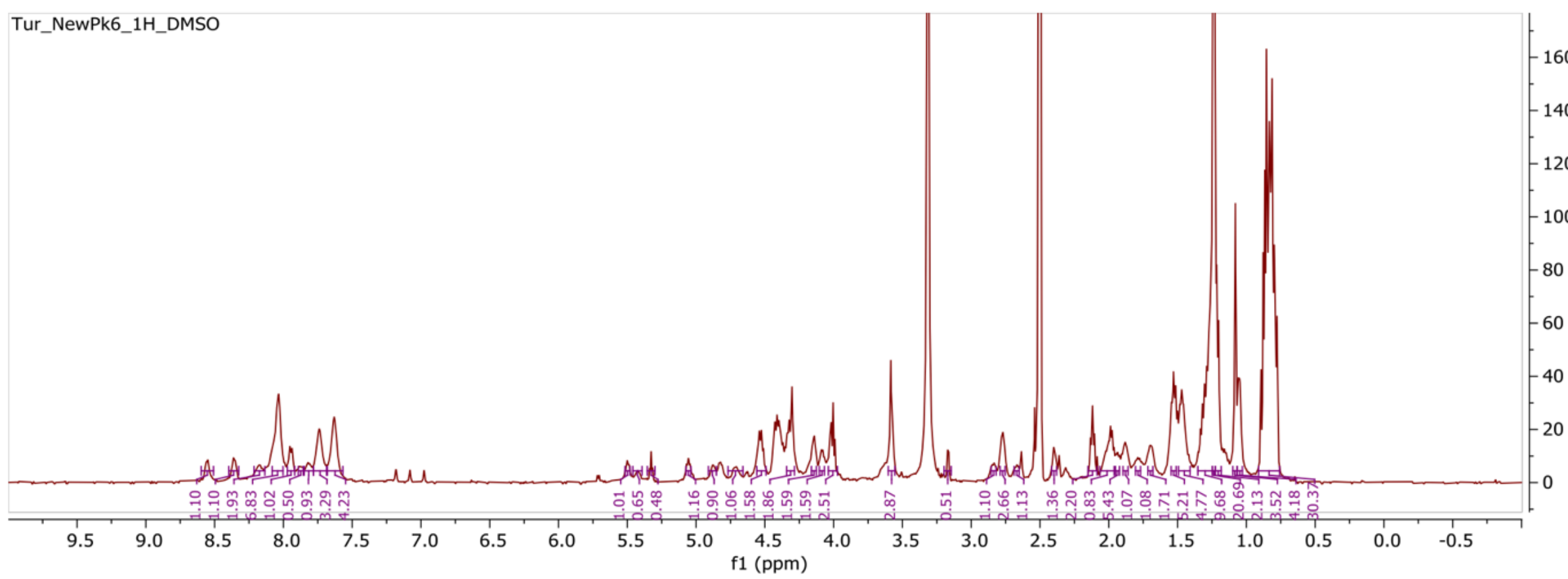

Figure S23. ^{1}H NMR spectrum of turnercyclamycin A butyl ester (**1b**) in DMSO-d_6 (500 MHz).

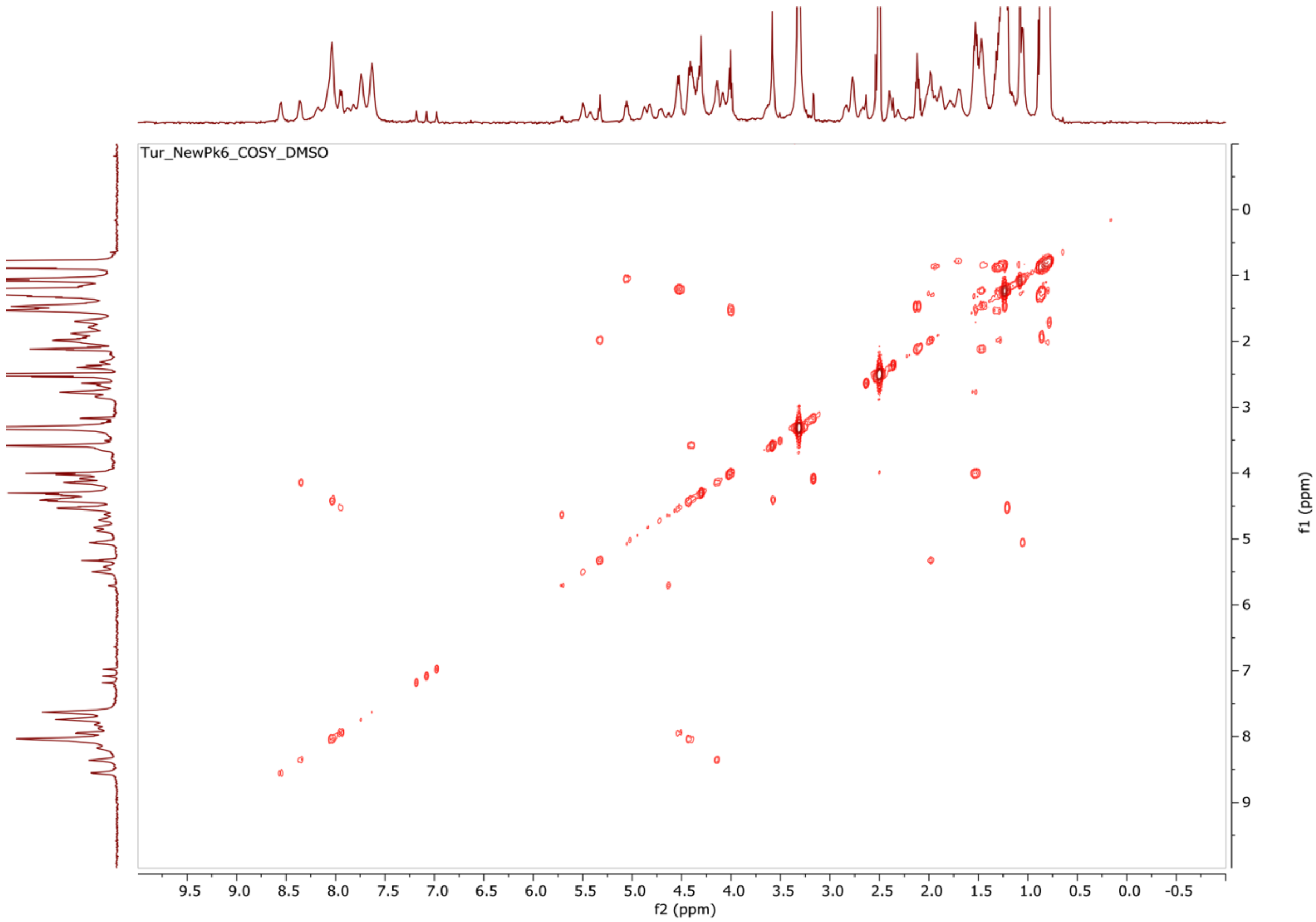

Figure S24. COSY NMR spectrum of turnercyclamycin A butyl ester (**1b**) in DMSO-d_6 (500 MHz).

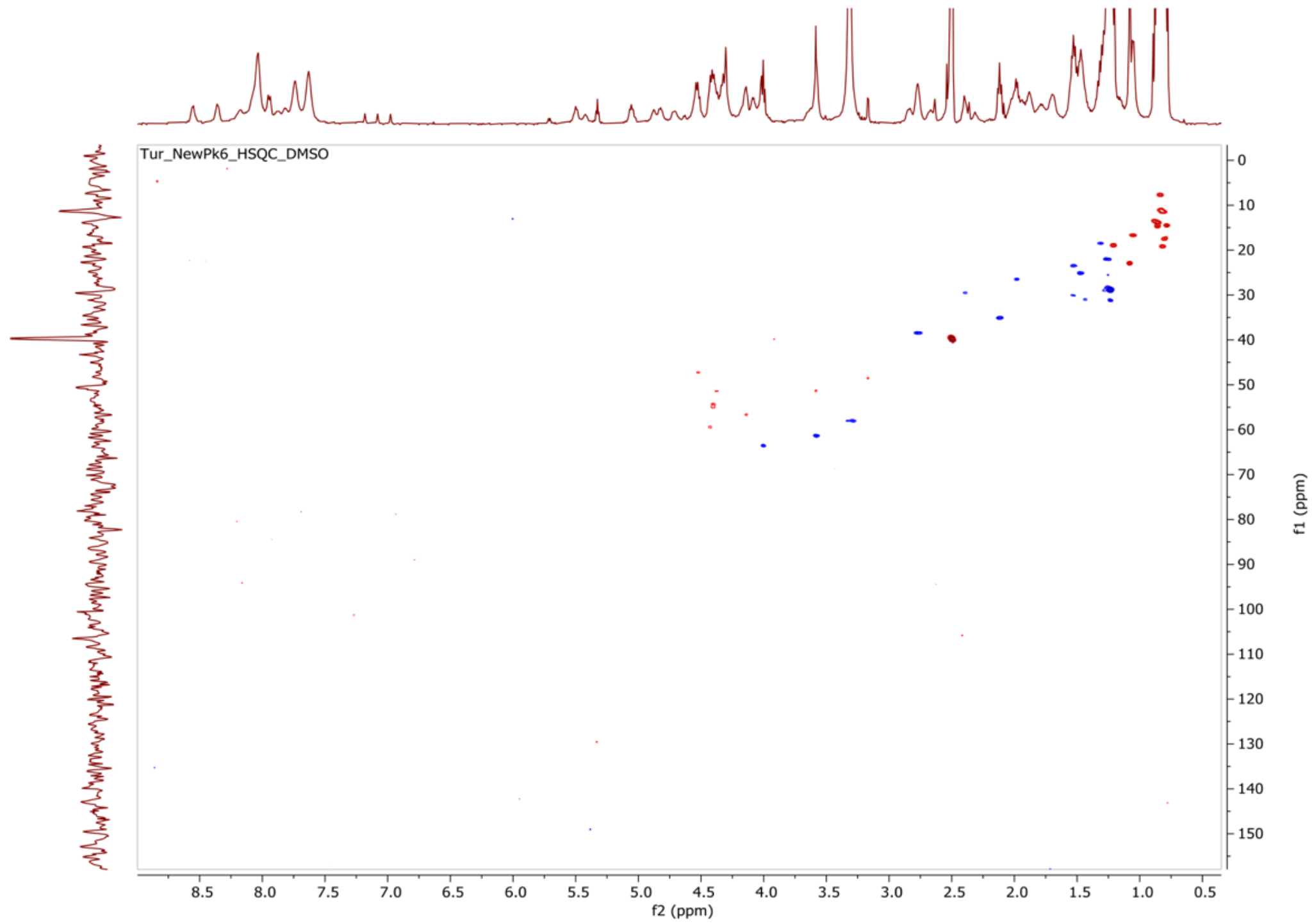

Figure S25. HSQC NMR spectrum of turnercyclamycin A butyl ester (**1b**) in DMSO-d_6 (500 MHz).

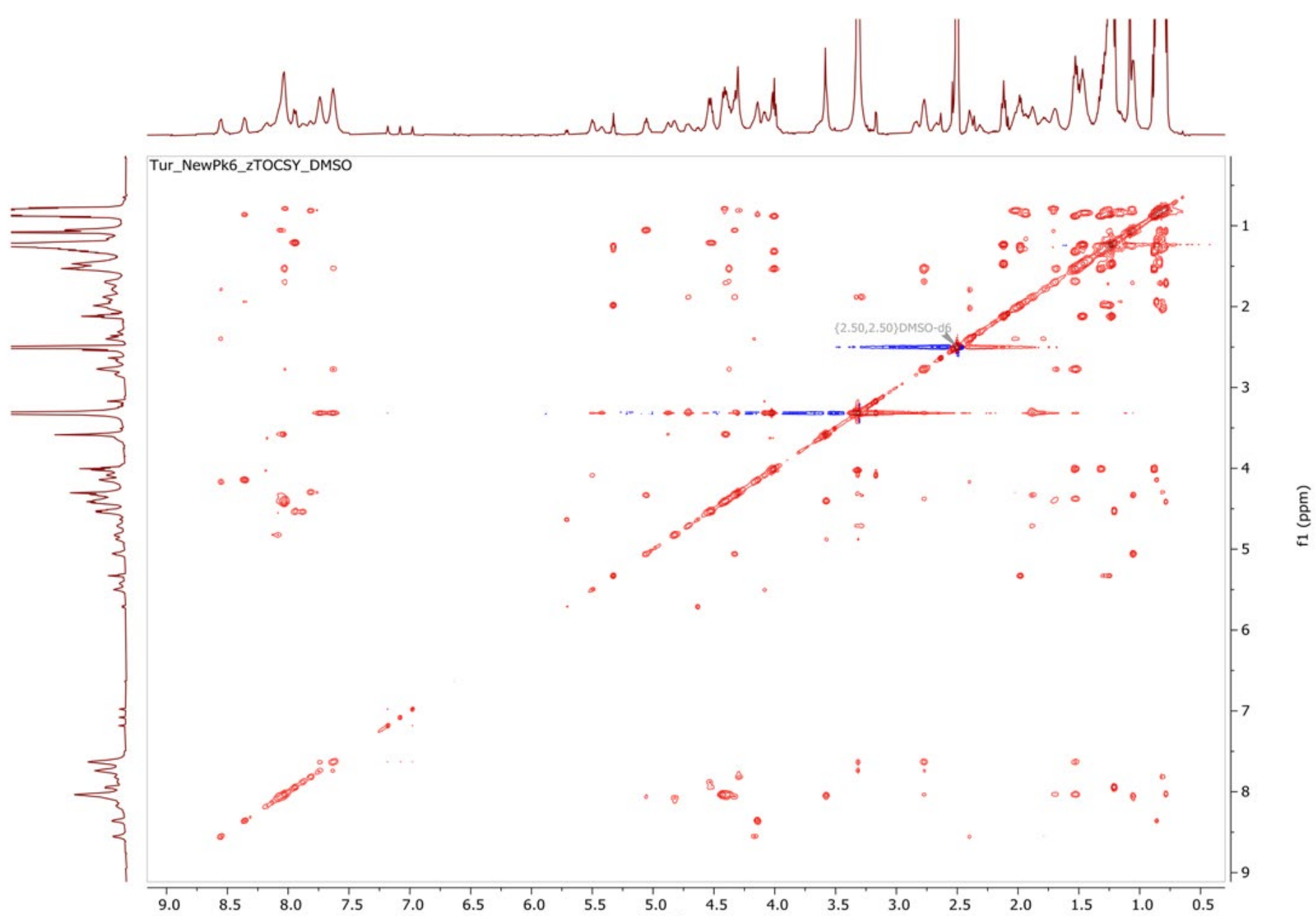

Figure S26. TOCSY NMR spectrum of turnercyclamycin A butyl ester (**1b**) in DMSO-d_6 (500 MHz).

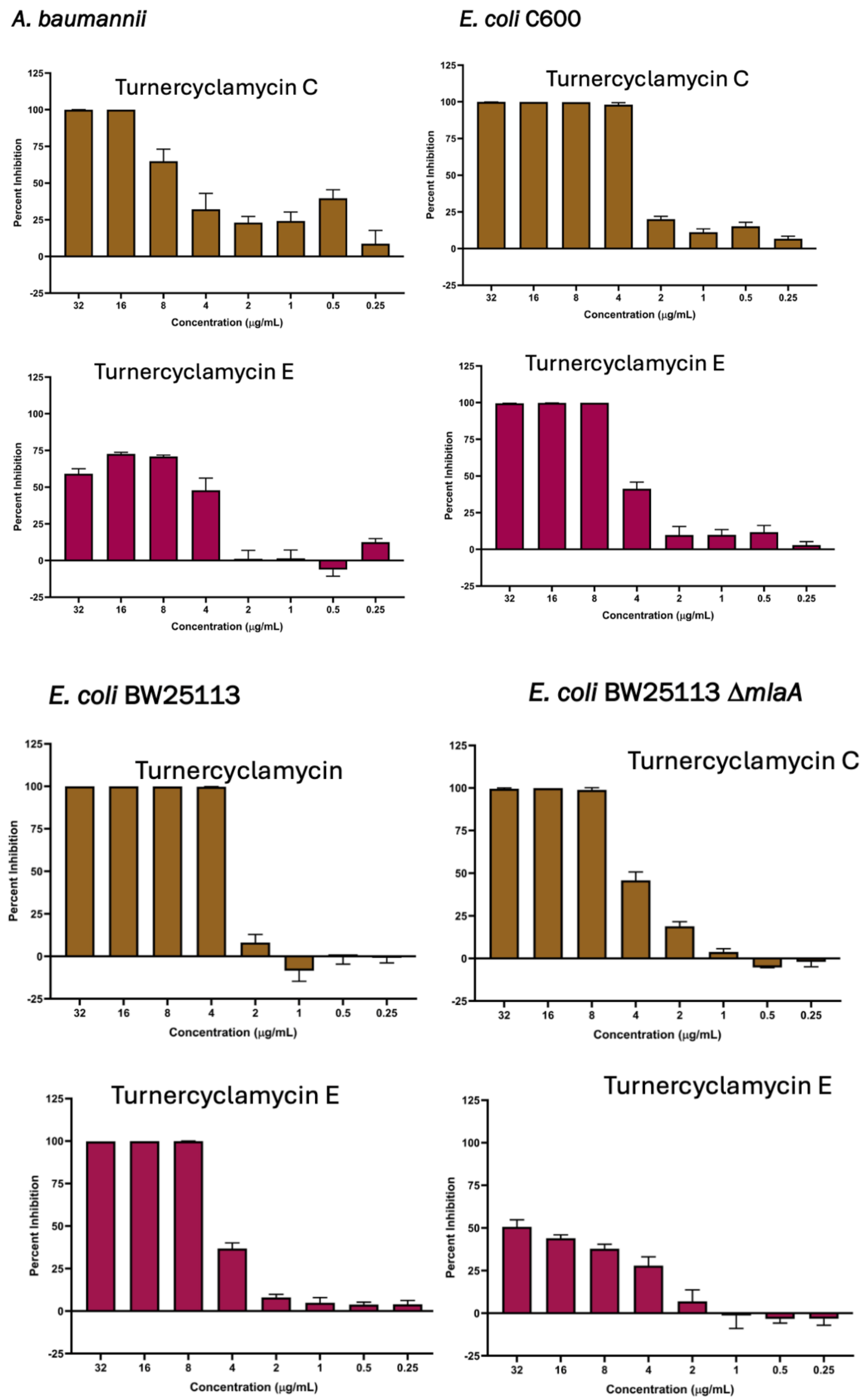

Figure S27. Raw results from antimicrobial assays.

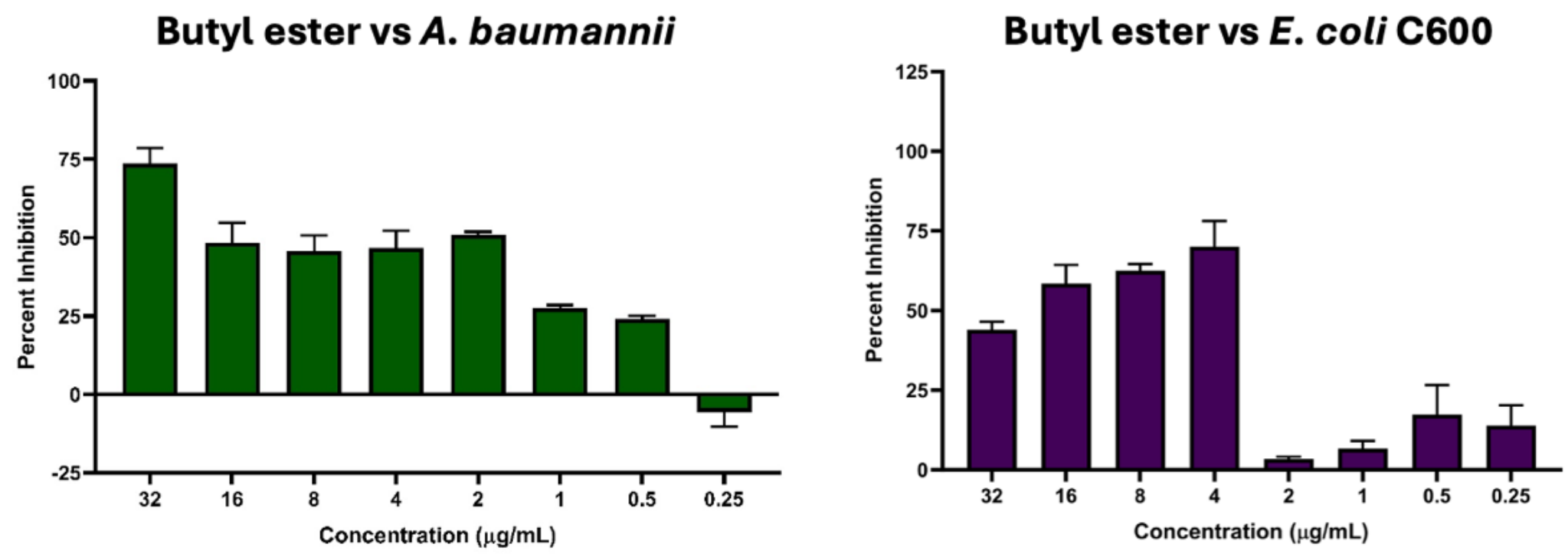

Figure S28. Butyl ester activity against bacteria; inhibition is observed, but no MIC_{90} or higher is obtained.

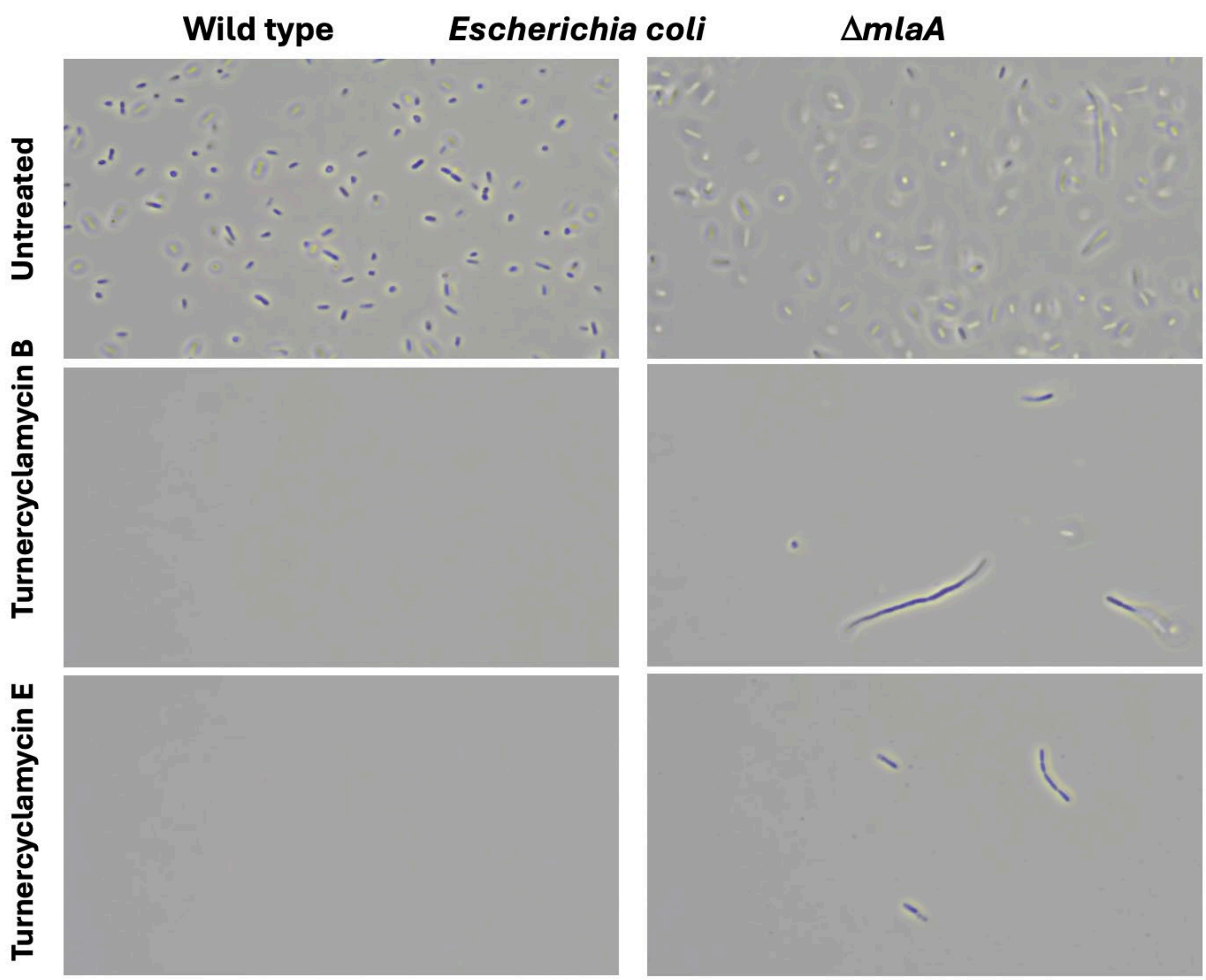

Figure S29. Light microscopy of *E. coli* BW25113 cells treated under different conditions. Wild type is killed by turnercyclamycins B and E, so that no cells are visible in the panel. At the same time point, both wild type and *mlaA* mutants that are not treated with antibiotics

show many small cells. By contrast, Δ*mlaA* treated with either antibiotic is resistant yet forms long filaments indicative of inhibition of cell division.

Table S1. Primers used in this study.

Primer Name	Sequence	Purpose
Down_FWD	atatgagtaaacttggtctgacagttaccaGTTCCGTATGGACCGACTTTC	Amplify downstream homology sequence
Down_REV	ggaatataagttatgGTTTACGTCACAATGACGTAGC	Amplify downstream homology sequence
Up_FWD	cattgtgacgtaaacCATAACTTATATTCCTTGGTCGCG	Amplify upstream homology sequence
Up_REV	cgtgcatcacgacaccgacaacctgcacatGGATATTCGTGTTAGCACTACG	Amplify upstream homology sequence
turH_KO_Seq1_FWD	ccataacgccgatgctgaaagc	Sequencing junction in KO plasmid
turH_KO_Seq1_REV	cctcaggccgggagatttattg	Sequencing junction in KO plasmid
turH_KO_Seq2_FWD	caccaaacaaaaccacgttgg	Verifying sequence in *T. turnerae* KO
turH_KO_Seq2_REV	gattatttcagagacttgggcg	Verifying sequence in *T. turnerae* KO
Sequence in red is the overhanging region for Gibson assembly		

Table S2. NMR chemical shift assignments compared to literature values for **2**

Residue	Position	Turnercyclamycin B **(2)**[b]		Turnercyclamycin A1 **(5)**		Turnercyclamycin C **(3)**	
		DMSO-d_6		DMSO-d_6		Methanol-d_4	
		δ_C	δ_H (J in Hz)	δ_C	δ_H (J in Hz)	δ_C	δ_H (J in Hz)
Ornithine	NH		8.03[a]		7.96[a]		
	C=O	171.6		171.6			
	α-C	51.5	4.37 m	51.5	4.37 m	53.6	4.47[a]
	β-C	29.0	1.53 m, 1.69 m	28.9	1.53[a], 1.71 m	29.5	1.72[a], 1.91[a]
	γ-C	23.6	1.53[a]	23.5	1.54[a]	24.8	1.74 m
	δ-C	38.5	2.77 br	38.5	2.78 br	40.1	2.99, t (6.9)
	NH_3		7.72		7.70 br		
Serine	NH		8.06[a]		8.02[a]		
	C=O	170.2		170.1		172.9	
	α-C	54.5	4.39[a]	54.4	4.4[a]	55.9	4.52[a]
	β-C	61.4	3.59 d (5.8)	61.4	3.59 d (5.7)	62.6	3.80 ddd (5.7, 11.5. 15)

	β-OH		N/A				
Valine	NH		7.82[a]		7.79		
	C=O	171.1		171.0		174.4	
	α-C	57.7	4.28	57.7	4.29[a]	61.5	4.1 td (6.5, 3.2)
	β-C	30.6	2.02 m	30.4	2.04 m	30.8	2.07[a]
	γ_1-C	17.6	0.80[a]	17.5	0.82[a]	19.2	1.02 d (6.7)
	γ_2-C	19.3	0.82[a]	19.1	0.83[a]	19.2	0.95[a]
β-OH-Aspartic acid	NH		8.13 d (9.0)		8.06		
	C=O	169.2		169.2		171.6	
	α-C	55.3	4.82 dd (8.8, 2.6)	55.3	4.82 dd (8.9, 2.7)	57.5	4.97, d (2.9)
	β-C	70.3	4.55[a]	70.2	4.55[a]	70.8	4.86[a]
	β-OH		N/A				
	γ-C = O	168.5		168.4[c]		174.6	
2,4-Diamino-3-hydroxybutyric acid	NH		7.85[a]		7.84[a]		
	C=O	172.8		172.7		170.8	
	α-C	55.1	4.55[a]	55.0	4.56 m	57.5	4.60 d (5.2)
	β-C	68.0	4.08 m	68.0	4.1 br	68.1	4.42 m
	β-OH		5.52 br		5.50 br		
	γ-C	41.7	2.67, 2.86 m	41.6	2.69, 2.86 br	43.1	3.02 dd (3, 12.8), 3.16 dd (3.3, 13)
	γ-NH_3		7.79 t (br)		7.78 br		
Threonine	NH		8.07[a]		8.04[a]		
	C=O	168.7		168.7[c]		170.9	
	α-C	56.6	4.32 m	56.5	4.34 m	58.0	4.61 d (5.7)
	β-C	70.2	5.06 p (6.7)	70.1	5.07 p (6.6)	71.8	5.32 p (7.0)
	γ-C	16.8	1.05 d (6.5)	16.6	1.07 d (6.5)	16.8	1.29[a]
Glycine	NH		8.19 t (6.1)		8.15 t (6.0)		
	C=O	169.4				171.1	
	α-C	41.3	3.6 m, 4.04 dd (16, 5.4)	41.3	3.64 m, 4.01 m	43.1	3.97 m
3-OH-isoleucine	NH		8.03[a]		7.96[a]		
	C=O	171.8		171.8		173.3	
	α-C	59.5	4.42 d (7.3)	59.3	4.45 d (6.4)	60.5	4.53[a]
	β-C	72.1		72.2		74.1	
	γ_1-C	23.0	1.08 s	23.0	1.09 s	23.3	1.23 s
	γ_2-C	31.1	1.45 m	30.9	1.45 m	32.2	1.56 q (7.6)
	δ-C	7.8	0.83[a]	7.7	0.84[a]	7.9	0.93[a]
Isoleucine	NH		8.36 d (7.8)		8.29 d (7.8)		

	C=O	170.7		170.7[c]		173.5	
	α-C	56.7	4.14 dd (7.6, 4.6)	56.6	4.17 dd (7.6, 4.6)	58.8	4.32[a]
	β-C	35.4	1.93 m	35.5	1.94 m	37.0	2.05 m
	γ_1-C	14.8	0.87 d (6.8)	14.6	0.86 d (7.2)	14.9	0.97 d (7.0)
	γ_2-C	25.7	1.16 m, 1.29 m	25.6	1.16 m, 1.30[a]	27.0	1.23[a], 1.45 m
	δ-C	11.6	0.81[a]	11.5	0.82[a]	11.6	0.94[a]
Homoserine	NH		7.61 d (br)		7.63 d (7.8)		
	C=O	171.1		171.2[c]		173.5	
	α-C	49.6	4.32[a]	49.7	4.32[a]	51.6	4.51[a]
	β-C	34.5	1.89 m	34.5	1.88 m	35.1	1.88 m, 2.09 m
	γ-C	58.1	3.31 m	57.9	3.32 m	59.4	3.56 m
	γ-OH						
Alanine	NH		7.94 d (8.6)		7.90[a]		
	C=O	172.4		172.2		174.7	
	α-C	47.4	4.52 m	47.4	4.49 q (7.4)	50.2	4.49[a]
	β-C	19.0	1.2 d (7.1)	18.7	1.22 d (7.1)	18.0	1.39 d (7.1)
Isoleucine (2) / Valine (2)	NH		8.01[a]		7.93[a]		
	C=O	171.5		171.2c		173.7	
	α-C	55.0	4.41[a]	57.1	4.25[a]	57.8	4.37[a]
	β-C	37.0	1.71 m	30.6	1.89 m	37.9	1.94[a]
	γ_1-C	25.6	1.06[a], 1.25[a]	18.1	0.82[a]	26.8	1.22[a], 1.43[a]
	γ_2-C	14.6	0.78 d (7.8)	18.1	0.82[a]	14.7	0.93[a]
	δ-C	11.1	0.83[a]			11.6	0.92[a]
Glutamic acid	NH		8.54 d (6.7)		8.47 d (7.1)		
	C=O	170.7		170.6[c]		171.9	
	α-C	52.5	4.17 m	52.2	4.22[a]	53.6	4.41[a]
	β-C	25.1	1.76 m, 1.99 m	25.3	1.78 m, 1.99[a]	26.6	1.97 m, 2.22 m
	γ-C	29.8	2.32 t (7.4)	29.7	2.32 t (7.5)	30.9	2.44 m
	δ-C = O	174.1		173.9		176.2	
Fatty Acid	1	172.4		172.4		176.2	
	2	35.1	2.12 t (7.1)	35.1	2.12 t (7.4)	36.5	2.25 dd (8.6, 6.2)
	3	25.3	1.47 m	25	1.49 m	26.6	1.61 m
	4	28.7-29.1	1.21-1.30	28.2-28.9	1.21-1.30	30.1	1.32[a]
	5	28.7-29.1	1.21-1.30	28.2-28.9	1.21-1.30	29.6-30.8	1.27-1.35
	6	28.7-29.1	1.21-1.30	28.2-28.9	1.21-1.30	29.6-30.8	1.27-1.35
	7	29.2	1.29	28.2-28.9	1.21-1.30	29.6-30.8	1.27-1.35

	8	26.7	1.98	26.5	1.98[a]	29.6-30.8	1.27-1.35
	9	129.6	5.32	129.6	5.33 t (5.2)	29.6-30.8	1.27-1.35
	10	129.6	5.32	129.6	5.33 t (5.2)	32.6	1.29[a]
	11	26.7	1.98	26.5	1.98[a]	23.4	1.31[a]
	12	29.2	1.29	31.1	1.25	14.2	0.9 t (6.7)
	13	28.7-29.1	1.21-1.30	21.9	1.27		
	14	31.1	1.24	13.8	0.87		
	15	22.1	1.26				
	16	13.9	0.85				

[a]multiplicity and coupling undetermined due to signal overlap

[b]Values from Miller et al 2021[12]

[c]No HMBC correlation, assigned based on comparison to **2**
